# Bacterial-type ferroxidase tunes iron-dependent phosphate sensing during *Arabidopsis* root development

**DOI:** 10.1016/j.cub.2022.04.005

**Published:** 2022-05-23

**Authors:** Christin Naumann, Marcus Heisters, Wolfgang Brandt, Philipp Janitza, Carolin Alfs, Nancy Tang, Alicia Toto Nienguesso, Jörg Ziegler, Richard Imre, Karl Mechtler, Yasin Dagdas, Wolfgang Hoehenwarter, Gary Sawers, Marcel Quint, Steffen Abel

**Affiliations:** 1 Department of Molecular Signal Processing, Leibniz Institute of Plant Biochemistry, Weinberg 3, 06120 Halle (Saale), Germany; 2 Department of Bioorganic Chemistry, Leibniz Institute of Plant Biochemistry, Weinberg 3, 06120 Halle (Saale), Germany; 3 Institute of Agricultural and Nutritional Sciences, Martin Luther University Halle-Wittenberg, Betty-Heimann-Strasse, 06120 Halle (Saale), Germany; 4 Gregor Mendel Institute of Molecular Plant Biology, Dr. Bohr Gasse 3, 1030 Vienna, Austria; 5 Research Institute of Molecular Pathology, Vienna BioCenter, Dr. Bohr Gasse 3, 1030 Vienna, Austria; 6 Proteome Analytics, Leibniz Institute of Plant Biochemistry, Weinberg 3, 06120 Halle (Saale), Germany; 7 Institute of Biology/Microbiology, Martin Luther University Halle-Wittenberg, Kurt-Mothes-Strasse 3, 06120 Halle (Saale), Germany; 8 Institute of Biochemistry and Biotechnology, Martin Luther University Halle-Wittenberg, Kurt-Mothes-Strasse 3, 06120 Halle (Saale), Germany; 9 Department of Plant Sciences, University of California, Davis, One Shields Avenue, Davis, CA 95616 USA; 10 German Center for Integrative Biodiversity Research, Halle-Jena-Leipzig, Puschstrasse 4, 04103 Leipzig, Germany

**Keywords:** Arabidopssis thaliana, root development, phosphate sensing, iron sensing, multicopper oxidase, plant ferroxidase, redox cycling, soil bacteria, horizontal gene transfer, plant terrestrialization

## Abstract

Access to inorganic phosphate (Pi), a principal intermediate of energy and nucleotide metabolism, profoundly affects cellular activities and plant performance. In most soils, antagonistic Pi-metal interactions restrict Pi bioavailability, which guides local root development to maximize Pi interception. Growing root tips scout the essential but immobile mineral nutrient; however, the mechanisms monitoring external Pi status are unknown. Here, we show that *Arabidopsis LOW PHOSPHATE ROOT 1* (*LPR1*), one key determinant of Fe-dependent Pi sensing in root meristems, encodes a novel ferroxidase of high substrate specificity and affinity (apparent *K*_M_ ∼ 2 μM Fe^2+^). LPR1 typifies an ancient, Fe-oxidizing multicopper protein family that evolved early upon bacterial land colonization. The ancestor of streptophyte algae and embryophytes (land plants) acquired LPR1-type ferroxidase from soil bacteria via horizontal gene transfer, a hypothesis supported by phylogenomics, homology modeling, and biochemistry. Our molecular and kinetic data on LPR1 regulation indicate that Pi-dependent Fe substrate availability determines LPR1 activity and function. Guided by the metabolic lifestyle of extant sister bacterial genera, we propose that *Arabidopsis* LPR1 monitors subtle concentration differentials of external Fe availability as a Pi-dependent cue to adjust root meristem maintenance via Fe redox signaling and cell wall modification. We further hypothesize that the acquisition of bacterial LPR1-type ferroxidase by embryophyte progenitors facilitated the evolution of local Pi sensing and acquisition during plant terrestrialization.

## Highlights

• *Arabidopsis thaliana* LPR1 multicopper oxidase typifies a novel ferroxidase cohort
• Fe availability tunes LPR1-dependent root responses to phosphate (Pi) limitation
• LPR1 specificity links Fe-Pi interactions to root Pi sensing via redox cycling
• Streptophyte ancestors acquired LPR1-type ferroxidase from soil bacteria by HGT

## Graphical abstract

**Figure:**
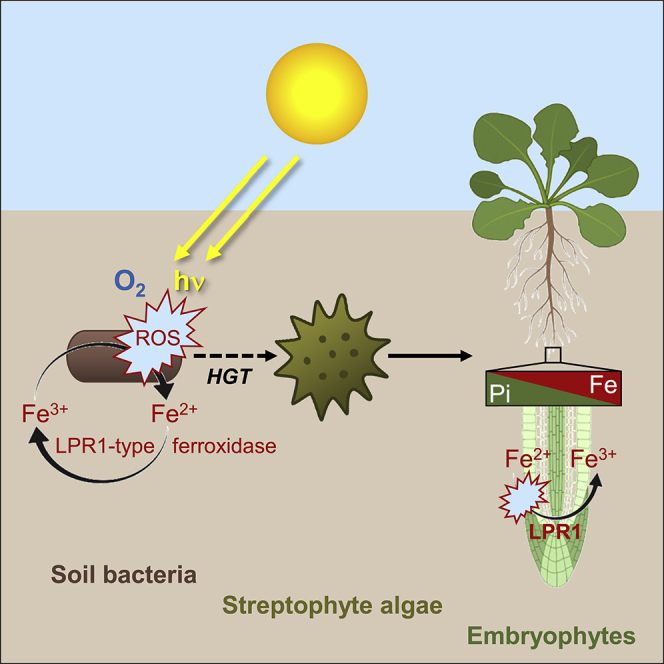

## Teaser

Phosphate (Pi) availability guides root growth. Naumann et al. show *Arabidopsis* LPR1 typifies a novel ferroxidase that links antagonistic Fe-Pi interactions to local Pi sensing by root tips via Fe redox cycling. Streptophyte ancestors gained LPR1-type ferroxidase from soil bacteria by horizontal gene transfer, which benefited plant terrestrialization.

## Introduction

Optimal plant growth critically depends on numerous edaphic resources. The central role of inorganic phosphate (H_2_PO_4_^−^ or Pi) in metabolism, paired with its scarce bioavailability, renders the mineral nutrient a highly limiting factor (together with N) of terrestrial primary production.^1^, ^2^, ^3^ Insolubility of most Pi salts and immobility of Pi complexed on clay mineral or metal oxide surfaces severely restrict biologic P accessibility. Thus, plants actively seek and mine the vital element and navigate Pi-associated metal (foremost Al and Fe) toxicities by adjusting root system architecture and modifying rhizosphere chemistry.^4^, ^5^, ^6^, ^7^, ^8^ When challenged by Pi limitation, most dicotyledonous plants attenuate primary root extension, stimulate lateral root proliferation, and promote root hair formation to increase the foraged soil volume.^8^, ^9^, ^10^, ^11^ To expedite Pi acquisition, coordinated biochemical processes release phospho-hydrolases and metal-chelating ligands (e.g., malate or citrate) into the rhizosphere to mobilize Pi from organic and mineral sources for efficient uptake.^5^^,^^8^ The numerous root tips formed are hotspots for Pi capture^12^ and monitor external Pi availability (local Pi sensing) to guide root development.^2^^,^^4^^,^^7^ On the other hand, profound metabolic adjustments that reprioritize organismic Pi utilization depend on internal Pi status (systemic Pi sensing) and are controlled by PHOSPHATE STARVATION RESPONSE 1 (PHR1) and PHR1-like MYELOBLASTOSIS (MYB)-related transcription factors.^13^^,^^14^

In *Arabidopsis thaliana* roots, Pi deprivation rapidly attenuates cell elongation in the transition zone (<2 h) and progressively inhibits cell division in the meristem (<2 days), growth processes typically monitored in primary roots.^15^^,^^16^ Persistent Pi starvation corrupts the stem cell niche (SCN) of the root apical meristem (RAM), followed by loss of RAM maintenance and ultimately by root growth arrest.^17^^,^^18^ Notably, local Pi sensing depends on external Fe availability, which points to antagonistic biologic Pi–Fe interactions.^15^^,^^16^^,^^18^, ^19^, ^20^, ^21^, ^22^, ^23^, ^24^, ^25^, ^26^ Genetic approaches identified several key components related to local root Pi sensing,^2^^,^^4^^,^^7^ including two functionally interacting genes expressed in overlapping cell types of root tips, *LOW PHOSPHATE ROOT 1* (*LPR1*) and *PHOSPHATE DEFICIENCY RESPONSE 2* (*PDR2*), which encode proteins of the secretory pathway.^18^^,^^20^ LPR1, a multicopper oxidase (MCO) with presumed Fe^2+^-oxidizing activity, is targeted to cell walls,^16^ whereas PDR2, the single P5-type ATPase, AtP5A in *Arabidopsis*, functions in the endoplasmic reticulum.^27^, ^28^, ^29^, ^30^ Upon Pi limitation, the LPR1-PDR2 module facilitates cell-type-specific Fe^3+^ accumulation in the apoplast, formation of reactive oxygen species (ROS), and cell wall modifications, which inhibit intercellular communication and thus root extension.^15^^,^^16^^,^^31^ Although ROS generation promotes peroxidase-dependent cell wall stiffening in the transition zone,^8^ callose deposition interferes with cell-to-cell communication and RAM activity.^9^ The current evidence points to LPR1 as one key component of Fe-dependent low-Pi sensing. Upon Pi deprivation, insensitive *lpr1* mutations cause unrestricted primary root extension by preventing Fe accumulation and callose deposition in root tips.^15^^,^^16^ Because loss of *LPR1* or external Fe withdrawal suppresses the hypersensitive *pdr2* root phenotypes in Pi deficiency, PDR2/AtP5A restricts LPR1 function by yet unknown processes.^16^^,^^24^^,^^28^ Thus, the elusive biochemical identity of LPR1 and the mechanism of LPR1 activation on low Pi need to be established.

Here, we show that *Arabidopsis LPR1* encodes a novel and prototypical MCO ferroxidase of bacterial origin. Although *LPR1* expression in root meristems is independent of external Pi or Fe status, and of *PDR2* function, LPR1 ferroxidase activity and LPR1-dependent root growth inhibition in limiting Pi are highly sensitive to low micromolar Fe concentrations, which represents a Pi-dependent cue monitored by root tips. Thus, Pi-dependent Fe substrate availability largely governs LPR1 function, whereas PDR2/AtP5A maintains Fe homeostasis of LPR1 reactants in root meristems. LPR1-related ferroxidases possess in their active site a distinctive acidic triad and flexible loop for high-affinity Fe^2+^-binding. Intriguingly, in addition to all extant land plants, LPR1-like proteins occur in streptophyte algae (Zygnematophyceae) and soil bacteria. Our phylogenetic and biochemical analyses support the hypothesis that LPR1-type MCO ferroxidases evolved early during bacterial land colonization and appeared in embryophytes (land plants) via horizontal gene transfer (HGT) from soil bacteria to the common ancestor of streptophytes (streptophytic algae plus embryophytes). Thus, the acquisition of a bacterial ferroxidase likely facilitated the evolution of local Pi sensing and acquisition during plant terrestrialization.

## Results

### *Arabidopsis LPR1* encodes a novel high-affinity ferroxidase

**Figure 1:**
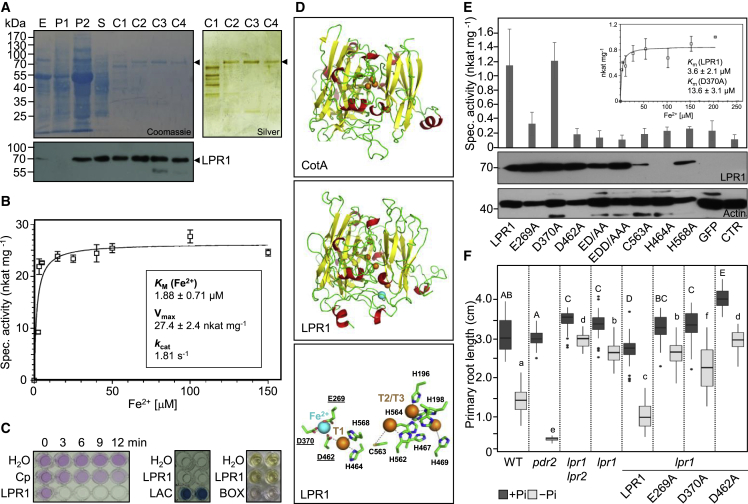
Properties of *Arabidopsis* LPR1 and structure-function analysis (A) Purification of native LPR1. Aliquots of fractions separated by SDS-PAGE (upper panels) and probed with anti-LPR1 (lower panel): leaf extract (E), ammonium sulfate precipitation (P), size-exclusion (S), and cation-exchange (C) chromatography. (B) Fe^2+^-dependent ferroxidase activity and catalytic constants of LPR1 (n = 4 independent LPR1 preparations). Shown is one representative of the four independent experiments; each assay performed in technical triplicates (±SD). (C) LPR1 substrate specificity. Ferroxidase assay (left): control, human ceruloplasmin (Cp). Lower intensity of Fe^2+^-ferrozine complex (pink) indicates Fe^3+^ formation. Laccase assay (center): control, laccase from *Trametes versicolor* (LAC). Colorless ABTS (2,2′-azino-bis[3-ethylbenzothiazoline-6-sulfonic acid]) oxidized to a blue product. Bilirubin oxidase assay (right): control, bilirubin oxidase from *Myrothecium verrucaria* (BOX). Decoloration indicates BOX activity. (D) LPR1 homology model. CotA structure PDB: 4AKP (upper panel) served as template to model LPR1 (center panel), accommodating the presumed four Cu sites (orange spheres) and Fe^2+^ substrate (blue sphere). Predicted Fe^2+^-binding site (E269, D370, and D462), proximal mononuclear T1 Cu site (H464 and H568), and distal trinuclear T2/T3 Cu cluster (lower panel). (E) Site-directed mutagenesis. Transient expression of LPR1 variants in tobacco leaves and specific ferroxidase activities (±SE; n ≥ 3). Controls: Mock (CTR) or *CaMV 35S*_*pro*_:*GFP* plasmid (GFP) infiltration. Inset: Fe^2+^-dependent specific ferroxidase activity of extracts expressing LPR1^WT^ (n = 3; shown are the combined values of three independent transformations, each including three technical replicates). Lower panel: immunoblot analysis of leaf extracts with anti-LPR1 and anti-actin. (F) Complementation of *lpr1* plants. Seedlings (+Pi, 5 d.a.g.) were transferred to +P or −Pi medium (25 μM Fe) and root extension was recorded 4 d.a.t. (±SD, n = 27–36 seedlings). Box plots show medians and interquartile ranges of gained root length; outliers (>1.5× interquartile range) are shown as black dots. Letters denote statistical differences in +Pi (capital) and −Pi (lower case) condition at p < 0.05 (one-way ANOVA and Tukey’s HSD post hoc test). See also Figures S1–S3 and Data S1.

To elucidate LPR1 identity, we purified to near homogeneity native LPR1 from stable transgenic (*CaMV 35S*_*pro*_*:LPR1*) *Arabidopsis* lines overexpressing LPR1 (STAR Methods). Immunoblot analysis, peptide sequencing, deglycosylation, and enzyme assays confirmed LPR1 preparations (∼70 kDa monomer), revealing no posttranslational modifications (Figures 1A, S1, and S2; Data S1). The MCO superfamily comprises four major classes, oxidizing substrates including polyphenols (laccases), bilirubin, ascorbate, and metals (e.g., yeast Fet3p ferroxidase^32^). Purified LPR1 exhibits Michaelis-Menten kinetics for high-affinity Fe^2+^ oxidation (apparent *K*_M_ ∼ 1.8 μM) and optimal activity at pH 5.8 (Figures 1B and S1I). Inability of LPR1 to oxidize representative substrates of each class, i.e., 2,2′-azino-bis[3-ethylbenzothiazoline-6-sulfonic acid] (ABTS, a laccase substrate), bilirubin, ascorbate, or manganese, supports Fe^2+^ specificity, whereas trivalent cations (Fe^3+^, Al^3+^, and Ga^3+^) are not effective inhibitors (Figures 1C and S3A–S3F). Thus, *Arabidopsis* LPR1 exhibits specific, high-affinity MCO ferroxidase activity.

We previously derived a structural model of LPR1 based on its low primary sequence identity with Fet3p (17%) and presumed function in root Fe homeostasis.^16^ Here, we identified high-scoring templates for homology modeling by position-specific iterative basic local alignment search tool (PSI-BLAST) iterations and Protein Data Bank (PDB) searches (STAR Methods). Surprisingly, the five best templates are experimental structures of spore-coat protein A (CotA), a bacterial (*Bacillus subtilis*) MCO laccase.^33^^,^^34^ The refined LPR1 model reveals the MCO hallmarks (T1 Cu site; T2/T3 Cu cluster) and predicts an acidic triad (E269, D370, and D462) for Fe^2+^-binding (Figures 1D and S3G), which we confirmed by site-directed mutagenesis and ferroxidase assays in transiently transgenic tobacco (*Nicotiana benthamiana*) leaves (Figure 1E). Ectopically expressed wild-type (WT) LPR1 showed the highest specific activity with an apparent *K*_M_ (3.6 μM Fe^2+^) similar to purified LPR1^WT^ (Figures 1B and 1E). Acidic triad mutations impaired ferroxidase activity to varying degrees. Although LPR1^D370A^ expression and activity were comparable to LPR1^WT^, LPR1^D370A^ showed about 4-fold lower Fe^2+^-affinity (apparent *K*_M_ ∼ 13.6 μM). LPR1^E269A^ transfection revealed ∼80% reduction of LPR1^WT^ specific activity above background, and LPR1^D462A^ expression was similar to control transfections. Variants with multiple substitutions (LPR1^E269A, D462A^ or LPR1^E269A, D370A, D462A^) or a compromised T1 site (LPR1^H464A^, LPR1^H568A^, or LPR1^C563A^) did not express ferroxidase activity above background. The latter variants were noticeably less abundant or undetectable (LPR1^H464A^), suggesting protein instability because Cu is a cofactor for MCO activity and folding (Figure 1E). ^35^

For verification *in planta*, we generated *lpr1* lines expressing LPR1^WT^ or LPR1 acidic triad variants. Comparison of root growth and Fe^3+^ accumulation in root tips upon seedling transfer to +Pi or −Pi medium supported our predictions (Figures 1F and S3H). Although LPR1^WT^ overexpression restored root growth inhibition of insensitive *lpr1* on −Pi agar, overexpression of LPR1^E269A^ or LPR1^D462A^ was ineffective, and LPR1^D370A^ complemented only poorly. Overall, the data are consistent with ferroxidase assays in tobacco. Finally, we purified LPR1 acidic triad variants, but none showed *in vitro* ferroxidase activity (Figures S3I and S3J). Thus, the predicted Fe^2+^-binding site is required for LPR1 ferroxidase activity (or stability) and LPR1-dependent Pi sensing processes by root tips.

### Fe availability tunes LPR1-dependent root responses to Pi deprivation

**Figure 2:**
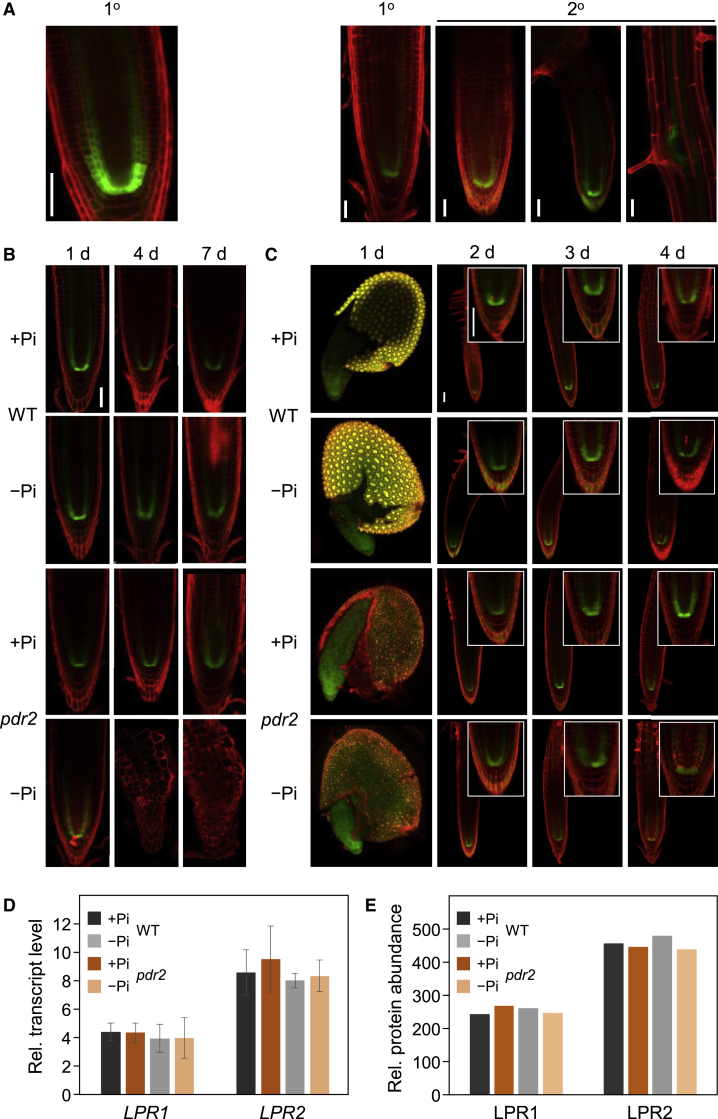
LPR1 expression in root meristems is independent of PDR2 and Pi availability (A) Expression of *LPR1*_*pro*_*:GFP* in primary (1°) and lateral (2°) root meristems of wild-type seedlings germinated on +Pi medium for 6 days (left panel) and 12 days (right panels). GFP fluorescence was analyzed in primary and lateral (different stages) meristems counterstained with PI (red). Representative images, n ≥ 7. Scale bars, 50 μm. (B) Expression of *LPR1*_*pro*_*:GFP* in primary root tips of wild-type (WT) and *pdr2* plants. Seedlings were germinated on +Pi agar (5 days) prior to transfer to +Pi or −Pi medium for up to another 7 days. Representative images, n ≥ 10. Scale bars, 50 μm. (C) Expression of *LPR1*_*pro*_*:GFP* in primary root tips of WT and *pdr2* seedlings germinated on +Pi or −Pi agar medium for up to 4 days. Representative images, n ≥ 10. z stack fusion of whole seed images. Scale bars, 50 μm. (D) Relative transcript levels (normalized to *UBC9*) of *LPR1*, *LPR2*, and *PDR2* in excised WT and *pdr2* root tips (no significant differences, two-tailed Student’s t test). Seeds were germinated on +Pi medium (5 days) and transferred to +Pi or −Pi medium. After 24 h, the root tip gain was harvested for RNA preparations (±SD; n = 3). (E) Relative protein abundance of LPR1 and LPR2 in excised root tips. Seeds were grown and root tips harvested as in (D) for quantitative proteomics by Tandem-Mass-Tag mass spectrometry. Shown is the sum of the normalized mass reporter intensities of LPR1 and LPR2 from one experiment. See also Figure S2.

To study LPR1 regulation, we analyzed *LPR1*_*pro*_*:GFP* expression in Pi-replete roots, revealing highest promoter activity in the SCN of primary and secondary meristems, with weaker GFP signals in proximal endodermal and cortical cells (Figure 2A). Upon seedling transfer to (or germination on) +Pi or −Pi medium, *LPR1*_*pro*_ activity in WT and *pdr2* root tips was similar on +Pi agar and not markedly changed in Pi-deprived WT (Figures 2B and 2C). GFP expression in Pi-deprived *pdr2* ceased 1 day after transfer (d.a.t.) due to RAM exhaustion. Because gene expression and RAM activity respond to Pi deprivation within 24 h,^16^^,^^24^ we monitored 1 d.a.t. *LPR1* mRNA in excised root tips. We also analyzed *LPR2*, the only *LPR1* paralog playing a minor additive role in local Pi sensing.^20^ Our data confirm *LPR1* and *LPR2* expression to be independent of Pi supply or *PDR2* function (Figure 2D). Although epitope-specific antibodies recognized LPR1 in roots of stable transgenic (*CaMV 35S*_*pro*_*:LPR1*) *Arabidopsis* plants, we did not detect LPR1 in WT or *pdr2* root extracts, even after profuse lateral meristem induction and LPR1 immuno- or chemical precipitation (Figures S2G–S2J). However, we confidently detected LPR1- and LPR2-derived peptides by quantitative proteomics in excised WT and *pdr2* root tips, indicating genotype- and Pi-independent abundance of LPR proteins in root meristems (Figure 2E).

**Figure 3:**
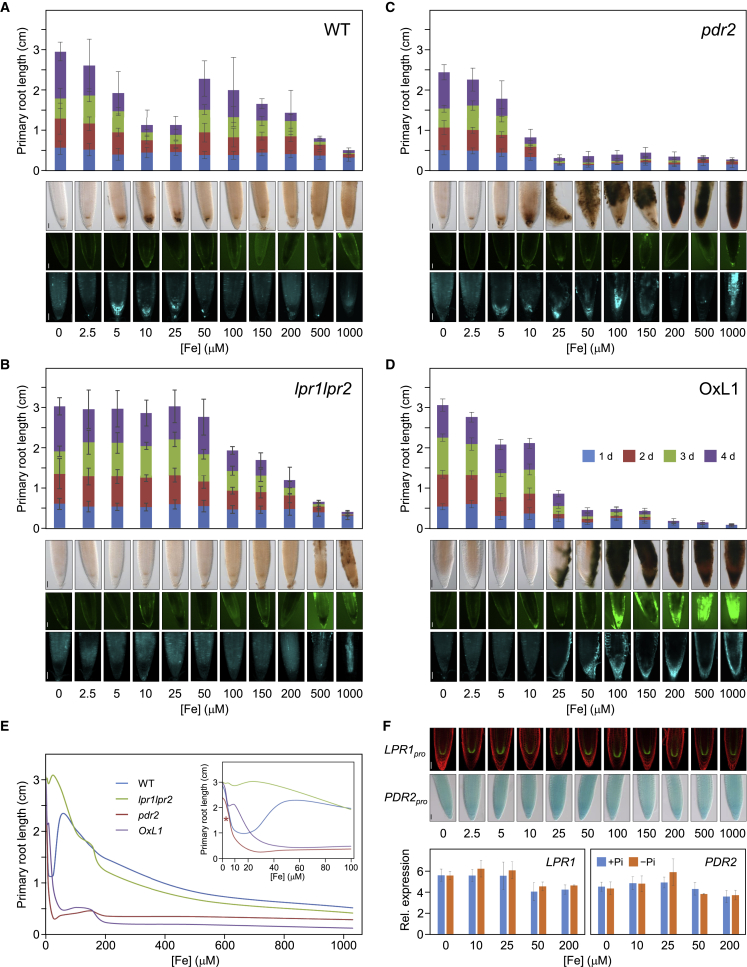
Increasing Fe availability in low Pi stimulates LPR1 function (A–D) Bar graphs: Fe-dependent inhibition of root growth on low Pi. Seeds of wild-type (A), *lpr1lpr2* (B), *pdr2* (C) and stable *CaMV 35S*_*pro*_*:LPR1* expressing (OxL1) plants (D) lines were germinated on +Pi agar (5 days) prior to transfer to −Pi media supplemented with increasing Fe^3+^-EDTA. Gain of primary root extension was daily recorded for up to 4 d.a.t. and plotted (±SD; n ≥ 50). Panels below bar graphs: for each Fe concentration, representative images (n ≥ 15) of Fe^3+^ accumulation (top row), ROS formation (center row), and callose deposition (bottom row) in Pi-deprived root tips. Fe^3+^ accumulation was monitored by Perls/DAB (3 d.a.t.), ROS formation by carboxy-H_2_DCFDA (1 d.a.t.), and callose deposition by aniline blue (3 d.a.t.) staining. Scale bars, 50 μm. (E) Trend lines of Fe-dependent root growth on −Pi agar. Inset: trend lines for the low Fe concentrations. Asterisk: *K_M_* (Fe^2+^) of LPR1. (F) Iron-independent *LPR1* and *PDR2* expression. Upper panel: transgenic *LPR1*_*pro*_*:GFP* and *PDR2*_*pro*_*:GUS* wild-type plants were grown as in (A)–(D). Representative images (n ≥ 15) of GFP and GUS expression (1 d.a.t.). Scale bars, 50 μm. Lower panel: relative *LPR1* and *PDR2* transcript levels. Root tip growth gain was harvested (1 d.a.t.) for RNA preparation and qRT-PCR analysis (±SD; n ≥ 3). See also Figure S4.

Next, we tested if substrate availability drives LPR1 function. We monitored root extension upon seedling transfer to agar with increasing Fe supply (0–1,000 μM). On +Pi/+Fe, root growth was comparable for all genotypes tested and not greatly altered up to 200 μM Fe, but it was inhibited at higher concentration due to Fe toxicity^36^ (Figure S4A). On −Pi/+Fe, we noticed striking genotype-dependent differences, with WT displaying a triphasic response (Figures 3A–3E). Low Fe (2.5–25 μM) gradually, but strongly, reduced root growth (by 60%), whereas intermediate Fe (50–100 μM) was less effective (30%), and high Fe (>100 μM) was as inhibitory as on +Pi medium. As expected, *lpr1lpr2* root growth was insensitive up to 50 μM Fe but was reduced on higher Fe. Roots of *pdr2* showed, as the WT, strong inhibition on low Fe (2.5–25 μM). Although reduction was maximal on 25 μM Fe (85%), higher Fe neither rescued nor intensified *pdr2* root inhibition. Remarkably, the apparent *K*_M_ (2 to 3 μM Fe^2+^) of LPR1 correlates with the initial (0–10 μM Fe) inhibition phase (compare Figures 1B, 1E, and 3E). Thus, because *LPR1* and *PDR2* expression does not respond to Fe supply (Figure 3F), substrate availability likely determines LPR1 activity in Pi limitation.

Because the *LPR1* expression domain overlaps with the RAM tissues accumulating Fe^3+^ on low Pi,^16^ we monitored Fe deposition in root tips (Figures 3A–3D). In Pi-deprived WT, Fe progressively accumulated in the SCN with increasing Fe supply. Staining intensity peaked at 10 μM Fe and decreased at higher Fe. The *lpr1lpr2* RAM did not accumulate Fe above background, except on high Fe (>500 μM). Cell-type specificity and intensity of Fe staining were similar between Pi-deprived WT and *pdr2* root tips on low Fe (<10 μM), whereas we noticed intense staining for *pdr2* on higher Fe. Upon transfer to +Pi/+Fe (Figure S4B), low Fe (0–10 μM) did not intensify Fe staining above background in any genotype. Moderate Fe (25–200 μM) appreciably increased Fe in the SCN and columella of WT and *pdr2*, but not *lpr1lpr2*, roots. High Fe caused Fe overload in all genotypes, indicating LPR1-independent root growth inhibition. ROS and callose production reflected Fe- and genotype-dependent patterns of Fe accumulation in Pi-deprived root tips (Figures 3A–3D). The similar response of WT and *pdr2* meristems to low Fe (0–10 μM) in terms of growth inhibition, Fe^3+^ accumulation, ROS formation, and callose deposition is consistent with the conclusion that PDR2 does not restrict LPR1 expression or activity. However, unrestrained Fe^3+^ accumulation in *pdr2* root tips at >10 μM Fe supply implicates PDR2 in maintaining Fe homeostasis (Figure 3C).

**Figure 4:**
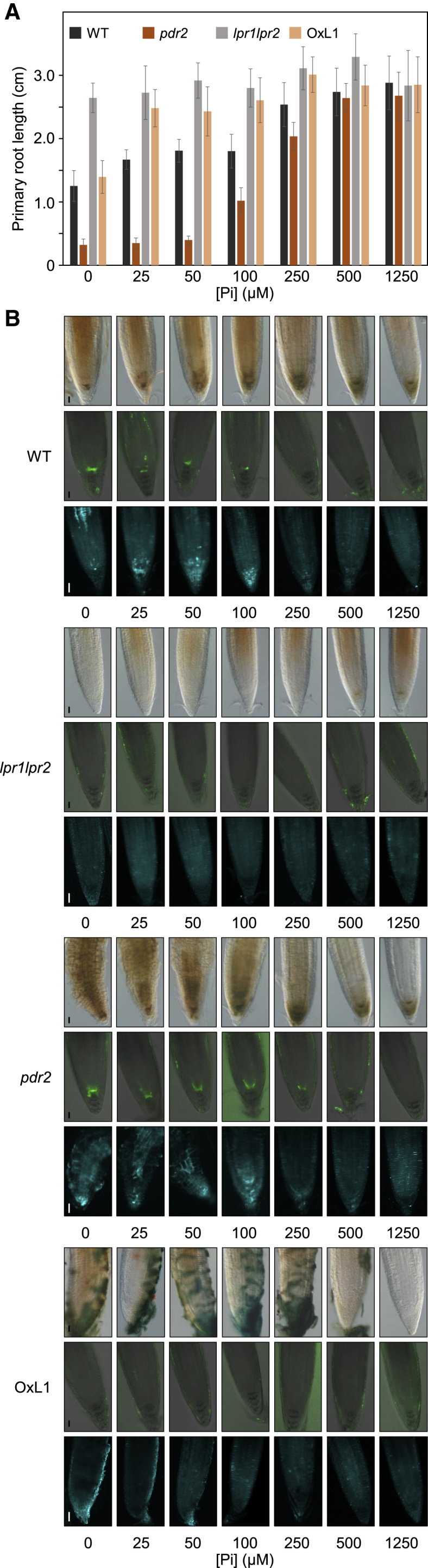
Increasing Pi availability in low Fe restricts LPR1 function (A) Pi-dependent rescue of root growth on low Fe. Seeds of the indicated genotypes were germinated on +Pi agar (5 days) prior to transfer to media supplemented with 25 μM Fe^3+^ and increasing Pi. Gain of primary root extension was measured (3 d.a.t.) and plotted (±SD; n ≥ 30). (B) For each genotype and Pi concentration, representative (n ≥ 15) images of Fe^3+^ accumulation (top rows), ROS formation (center rows), and callose deposition (bottom rows) in root tips (monitored as in Figures 3A–3D). Scale bars, 50 μm.

Finally, we studied root responses at 25 μM Fe (an effective inhibitory Fe concentration in low Pi; see Figure 3A) to increasing Pi supply (0–1,250 μM). Although root growth of insensitive *lpr1lpr2* was not affected, WT and *pdr2* seedlings responded progressively, attaining maximal root extension at 250 and 500 μM Pi, respectively (Figure 4A). Fe accumulation, ROS formation, and callose deposition correlated inversely with root extension and diminished in root tips on increasing Pi (Figure 4B). Thus, because Pi strongly complexes metal cations, Pi-dependent Fe availability determines LPR1 activity.

### LPR1 intersects with Fe redox cycling in low Pi

**Figure 5:**
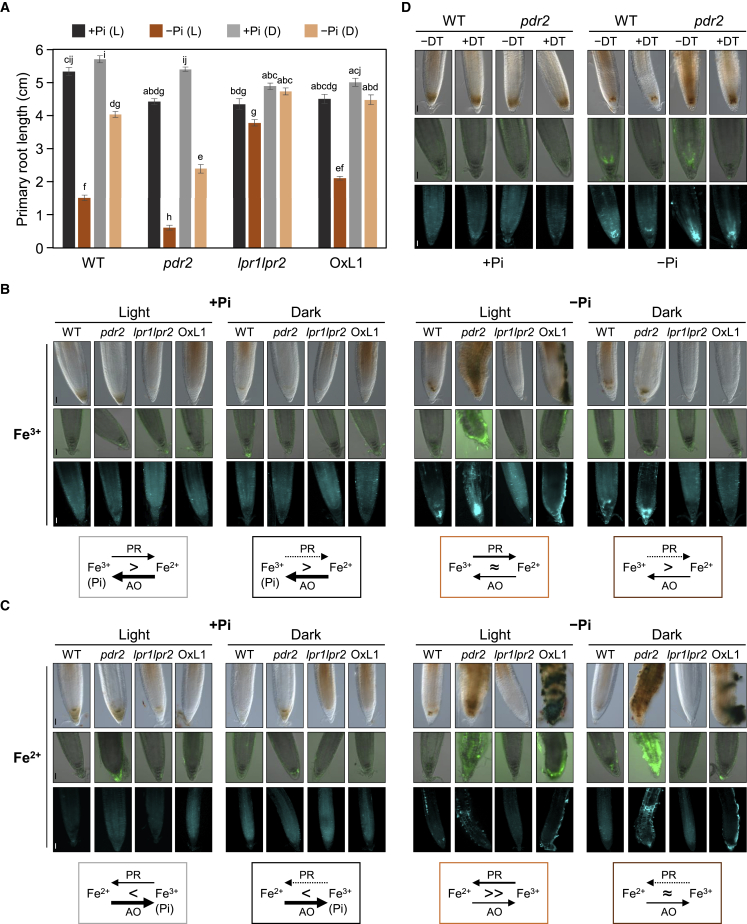
LPR1 intersects with Fe redox cycling in low Pi (A) Growth of illuminated (L) or light-protected (D) roots 6 d.a.t. of 5-day-old seedlings to +Pi or −Pi agar supplemented with 25 μM Fe^3+^ (±SD; n ≥ 30). Letters denote statistical differences at p < 0.05 (two-way ANOVA and Tukey’s HSD post hoc test). (B and C) Seeds were germinated on +Pi agar (5 days) prior to transfer to illuminated or partially shielded (roots), +Pi or −Pi agar medium supplemented with 25 μM Fe^3+^ (B) or 25 μM Fe^2+^ (C). Representative images (n ≥ 15) of Fe^3+^ accumulation (top row), ROS formation (center row), and callose deposition (bottom row) in root tips (3 d.a.t.) monitored as in Figures 3A–3D. Scale bars, 50 μm. The scheme below each condition indicates the prevalent Fe speciation, resulting from Fe^3+^ photoreduction (PR) and Fe^2+^ autoxidation (AO). (D) LPR1- and ROS-dependent callose deposition on illuminated +Pi or −Pi agar (25 μM Fe^3+^) with or without 750 μM DMTU (DT). Representative images (n ≥ 15) of Fe^3+^ accumulation (top row, 3 d.a.t.), ROS formation (center row, 1 d.a.t.), and callose deposition (bottom row, 3 d.a.t.) in root tips, monitored as in Figures 3A–3D. Scale bars, 50 μm. See also Figure S5.

To explore a presumed function of LPR1 in Fe redox cycling, we first compared Pi-dependent root growth on fully illuminated and partially shaded agar plates using modified D(dark)-Root devices^37^ (Figure S5A). Recent studies reported root illumination inhibits root extension on low Pi, likely caused by photochemical acceleration of Fe redox cycling and ROS formation.^38^^,^^39^ As expected, growth inhibition of shaded roots on −Pi agar was less severe when compared with illuminated roots. However, the genotype-specific differences in root growth (Figure 5A) and RAM responses (Figure 5B), including the nearly constitutive *LPR1* expression (Figures S5D–S5F), were maintained on −Pi agar, indicating LPR1-dependent processes operate in dark-grown roots.

We previously reported partial *LPR1* silencing in the RAM of stable transgenic (*CaMV 35S*_*pro*_*:LPR1*) plants but ectopic expression in root cap cells.^16^ Thus, roots of such plants allow assessment of LPR1 function *in situ*, i.e., at the columella-agar interface, as a proxy for the internal root apoplast. Because agar illumination promoted photo-Fenton Fe^3+^ reduction (Figure S5B) and ensuing Fe^2+^ autoxidation (AO),^40^, ^41^, ^42^ we estimated Fe speciation in +Pi and −Pi media (supplemented with Fe^3+^ or Fe^2+^) upon illumination or darkness (Figure S5C). Irrespective of the Fe source, Fe^3+^ dominate in all four +Pi conditions because Pi (a strong Fe^3+^-chelator) accelerates Fe^2+^ AO and slows Fe^3+^ photoreduction (PR).^42^^,^^43^ Significant Fe^2+^ was generated in illuminated −Pi/+Fe^3+^ medium, and maintained in light- or dark-exposed −Pi/+Fe^2+^ media (Figure S5C). Upon seedling transfer to Fe^2+^-sustaining media, root tips of stable transgenic (*CaMV 35S*_*pro*_*:LPR1*) plants showed Fe^3+^, ROS and callose formation in agar-contacting columella cells (Figures 3D, 5B, and 5C). Such formation was suppressed on +Pi and light-shielded −Pi/+Fe^3+^ media (Figures 4B, 5B, 5C, and S4), supporting *in situ* LPR1 ferroxidase activity. Importantly, in WT and *pdr2* root tips, the LPR1-dependent processes monitored can be uncoupled by dimethylthiourea (DMTU), a scavenger of hydroxyl radicals generated in Fe redox cycling.^44^^,^^45^ Although DMTU treatment did not abolish Fe^3+^ deposition, it noticeably reduced ROS and callose formation (Figure 5D), indicating disruption of Fe redox cycling in the native LPR1 domain and activation of callose deposition downstream of LPR1 function.

### *Arabidopsis* LPR1 typifies an ancient ferroxidase family

**Figure 6:**
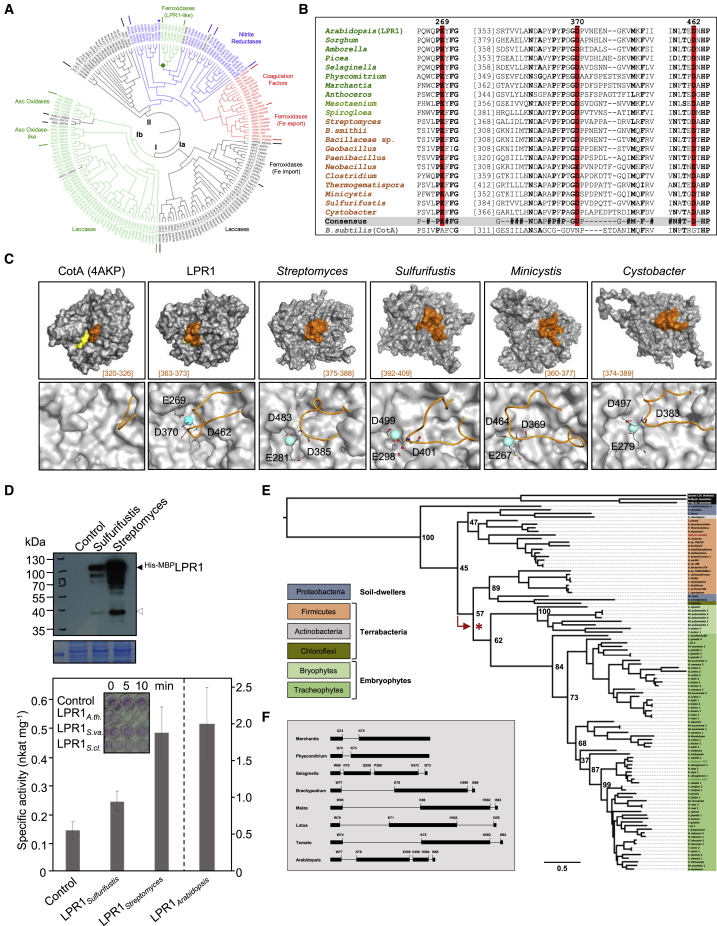
Land plant progenitors acquired LPR1-type ferroxidase from soil bacteria (A) Maximum-likelihood midpoint-rooted phylogenetic tree of 188 annotated multicopper oxidase (MCO) proteins (UniProt Knowledge Database). Group I: Fet3p-related ferroxidases and fungal laccases (clade Ia, gray); plant laccases and ascorbate oxidases (clade Ib, green). Group II: MCOs from archaea (black), bacteria (blue), and animals (red). CotA (^∗^) and LPR1-like proteins (green) are monophyletic within the paraphyletic segment of bacterial MCOs. (B) Alignment of conserved sequence motifs flanking each residue of the acidic triad relative to *Arabidopsis* LPR1 (E269, D370, D462, red; conserved residues, bold). The central residue (D370) is located in a variable linker terminated by conserved motifs. Alignment of select LPR1-like MCOs of plants (green), Zygnematophycea (light green), soil bacteria (brown), and CotA. (C) Experimental structure of *Bacillus* CotA (PDB:4AKP), homology models of *Arabidopsis* LPR1 and LPR1-like MCOs from *Streptomyces clavuligerus*, *Sulfurifustis variabilis*, *Minicystis rosea*, and *Cystobacter fuscus* (see Figure S7D for superposition of protein models). Upper row: surface representations highlight the ABTS-binding pocket (yellow) and adjacent short surface loop (orange) on CotA. Highlighted is the corresponding surface loops (orange) on each protein model (position of amino acid residues in brackets). Lower row: enlarged predicted substrate-binding sites depict the surface loop (orange ribbon), the acidic triad (sticks), and Fe^2+^ substrate (sphere). (D) Ferroxidase activity of bacterial LPR1-type MCOs. Upper panel: expression of affinity-tagged enzymes from *Sulfurifustis* and *Streptomyces* in *E. coli* (n = 4). Lower panel: specific ferroxidase activity of *E. coli* extracts after induction of recombinant protein expression (±SD, n = 3), and of transgenic (*CaMV 35S*_*pro*_*:LPR1*) *Arabidopsis* leaf extract. Inset: ferrozine assays with *E. coli* and leaf extracts. (E) Maximum-likelihood midpoint-rooted tree (400 bootstrap replicates) of select LPR1-like MCOs from bacteria and embryophytes (see Data S2A). The latter occupy a monophyletic clade nested within the bacterial radiation, suggesting a single HGT event (red arrow). (F) Gene models of select *LPR1*-like genes suggest acquisition of phase-0 introns (separating symmetric exons). See also Figures S6 and S7 and Data S2, S3, S4, and S5.

LPR1-like MCOs are ubiquitous in the embryophytes and encoded by small orthogroups (1–5 genes; Data S2A, S2C, and S4). The substantial primary structure identity between *Arabidopsis* LPR1 and *Bacillus* CotA (37%), approaching the similarity between LPR1 and bryophyte LPR1-like proteins (∼40%), prompted phylogenetic analysis of annotated MCOs (Figure 6A). Group I of the bifurcated cladogram contains fungal laccases and ferroxidases related to Fe import, plant laccases, and ascorbate oxidases. Group II comprises bacterial, fungal, and mammalian MCOs of unknown specificities or presumed functions in N assimilation (nitrite reductases), Fe export (ceruloplasmin-related ferroxidases), and hemostasis (blood coagulation factors). CotA and LPR1-like MCOs of *Arabidopsis* and rice^46^^,^^47^ occupy a monophyletic clade within the bacterial paraphyletic segment. Comparison of the primary and tertiary structures rationalizes the strikingly different substrate specificities of LPR1 and CotA, which oxidizes bulky molecules such as ABTS or bilirubin.^33^ Alignment of CotA and LPR1-like MCOs indicates absence of a *bona fide* Fe^2+^-binding acidic triad in CotA (Figure S6). Although E269 and D462 on LPR1 are each embedded in a conserved segment, D370 is located in a variable linker flanked by two hydrophobic motifs (Figure 6B). Although these features, but not the acidic triad residues, are conserved in CotA, its linker sequence is shorter (aa 321–326) and likely folds into a tight surface loop, permitting access of large substrates (Figures 6C and S7A). The longer surface loop on LPR1 (aa 363–373) harboring D370 may provide a lid-like latch for high-affinity Fe^2+^ binding (Figure 6C). Despite a similar configuration of the Fe^2+^-binding and electron-transfer sites near the T1 Cu center, the topology of the Fe^2+^-binding pocket greatly differs between LPR1 and yeast Fet3p (Figures S7B and S7C).^48^

A hidden Markov model (HMM) search indicated widespread occurrence of CotA-like MCOs in Bacteria and Archaea. If filtered for the LPR1-type acidic triad, we identified >35 LPR1-like proteins (Data S2, S3, and S4). Homology modeling of four such MCOs suggests the presence and topology of an LPR1-type surface loop for Fe^2+^-binding (Figure 6C). Indeed, when expressed in *Escherichia coli*, LPR1-like MCOs from *Streptomyces clavuligerus* and *Sulfurifustis variabilis* showed ferroxidase activity (Figure 6D). Bacterial LPR1-like MCOs are limited to five phyla (Data S2A), comprising so-called Terrabacteria (Firmicutes, Actinobacteria, and Chloroflexi), and soil-dwelling members of Bacteroidetes and Proteobacteria.^49^ A cladogram of LPR1-like MCOs of bacteria and embryophytes reveals a monophyletic clade of the plant sequences nested within the bacterial radiation (Figure 6E). The tree topology suggests a single HGT event from a bacterial donor to a land plant progenitor, which is supported by the increasing number of phase-0 introns during the evolution of *LPR1*-like genes in the embryophytes (Figure 6F). Phase-0 introns are thought to partition the acquired bacterial gene into symmetric exons for maintaining its ancient function.^50^ *LPR1*-like genes diversified by tandem duplication (e.g., rice^47^) or whole genome duplication (WGD). The syntenic gene pair, *LPR1* (At1g23010) and *LPR2* (At1g71040), likely originated from the α-WGD in the Brassicaceae approximately 32 million years ago (TAIR Synteny Viewer).^51^

**Figure 7:**
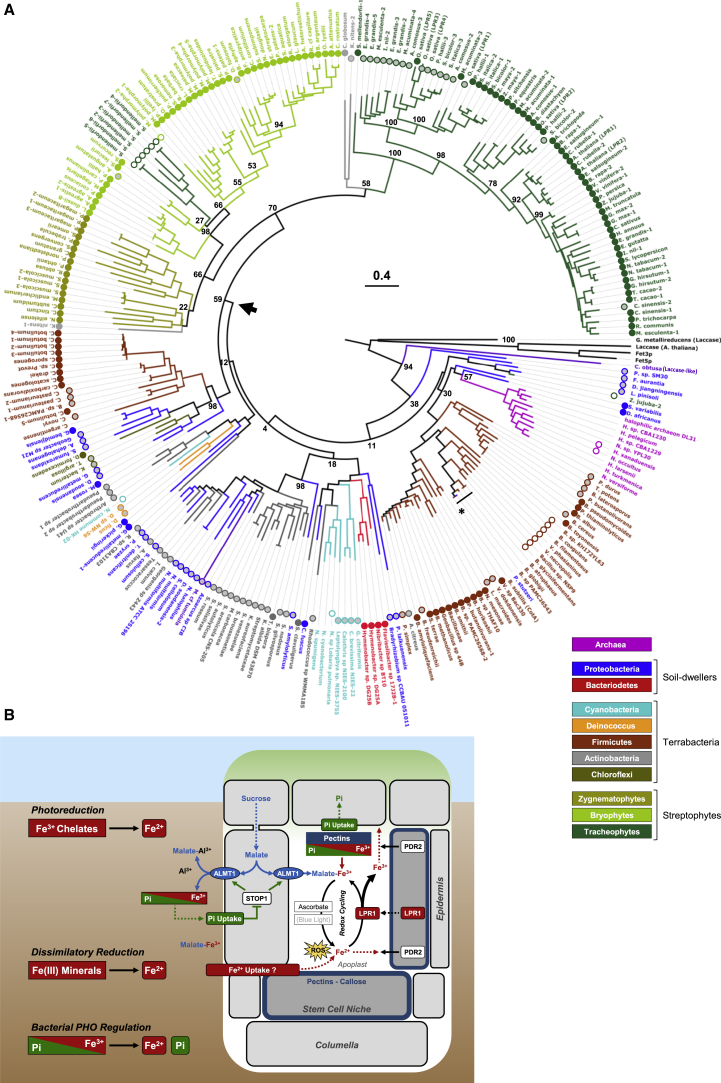
LPR1-type ferroxidases emerged during bacterial land colonization (A) Maximum-likelihood midpoint-rooted tree (250 bootstrap replicates) of predicted LPR1-like MCOs from bacteria, streptophyte algae and embryophytes. The presence of a complete (3/3) or partial acidic triad motif is indicated by circles next to each species (3/3—full color; 2/3—gray; 1/3—white; no circle—absence). CotA (*B. subtilis*) and highly similar CotA-like proteins are indicated by a black asterisk and bar, respectively. The streptophyte sequences occupy a monophyletic clade nested within the paraphyletic bacterial radiation, supporting a single HGT event from a bacterial donor (black arrowhead). The authenticity of the three sequences in the Klebsormidiophyceae and Coleochaetophyceae (gray), and of the single hit in the Chlorophyceae (purple) remain to be validated (STAR Methods). (B) Model of LPR1 function. ALMT1-mediated malate release into the rhizosphere and root apoplast chelates toxic Al^3+^ in soil and mobilizes Pi from Fe–Pi complexes by Fe^3+^-chelation. Ascorbate reduces Fe^3+^-malate, stimulating ROS formation by Fenton chemistry. LPR1-dependent Fe^2+^ oxidation attenuates ROS production and presumably ROS signaling in the stem cell niche. Fe^2+^ substrate availability tunes LPR1 ferroxidase activity. PDR2/AtP5A counteracts LPR1 function by maintaining Fe homeostasis in root tips. Fe^2+^ generation in the SCN apoplast, and possibly Fe^2+^ uptake from the rhizosphere into the columella/SCN apoplast, constitute a local cue for external Pi availability monitored by the PDR2-LPR1 module. In Pi limiting and sterile laboratory conditions, UV or blue light illumination stimulates ROS formation and root growth inhibition. In such settings, Fe^3+^ photoreduction likely mimics the impact of soil bacteria, which mobilize redox-active Fe^2+^ from Fe(III) oxide minerals by various metabolic processes. See also Data S2A, S2C, S4, and S5.

The embryophytes likely evolved from the streptophyte algae, which comprise five classes of freshwater and terrestrial algae (Mesostigmatophyceae/Chlorokybophyceae, Klebsormidiophyceae, Charophyceae, Coleochaetophyceae, and Zygnematophyceae).^52^ Phylogenomics favors the Zygnematophyceae or the Coleochaetophyceae/Zygnematophyceae clade as the sister group of land plants.^53^ We identified *LPR1*-like genes in the Zygnematophyceae and Klebsormidiophyceae, but not in the Charophyceae and Mesostigmatophyceae/Chlorokybophyceae.^54^^,^^55^, ^56^, ^57^, ^58^ HMM analysis of the One Thousand Plant Transcriptomes^59^ identified *LPR1*-like sequences in the bryophytes and streptophyte algae (23 Zygnematophyceae/1 Coleochaetophyceae), one weakly supported hit in the chlorophytes (115 species), and no hits in the glaucophytes and rhodophytes (Data S2, S3, and S4). A cladogram including the additional LPR1-like MCOs further supports HGT from a bacterial donor to a progenitor of the streptophytes (Figure 7A).

Finally, to explore the origin of bacterial LPR1-like ferroxidases, we searched for MCOs with incomplete acidic triad motifs. We identified >80 sequences, all limited to Terrabacteria, Proteobacteria, and Halobacteria (Data S2B, S2C, and S4). Most of the MCOs (∼60) lack the second conserved acidic residue in its linker (Data S3), which, however, is expendable for high-affinity Fe^2+^-binding (see Figure 1E). Members of the Firmicutes show the highest variation of partial acidic triads, suggesting CotA-type laccases evolved from LPR1-type MCOs by linker contraction and triad degeneration (Data S3). The cladogram of CotA- and LPR1-like bacterial MCOs suggests that LPR1-type ferroxidases arose early during bacterial land colonization and crossed phyla multiple times to diversify by HGT, which is widespread among soil bacteria^60^ (Figure 7A; Data S5).

## Discussion

Variable Pi availability in soil guides root development via local adjustment of root tip growth. When encountering Pi deprivation, the *LPR1-PDR2* module asserts genetic control of root meristem activity, which sensitively responds to co-occurring Fe as a Pi-dependent cue.^16^^,^^18^^,^^20^ Here, we show that LPR1, one key determinant of local Pi sensing in *Arabidopsis*, encodes a novel, prototypical MCO ferroxidase of high specificity and Fe^2+^ affinity (Figures 1 and S3). MCO proteins, widely distributed in all domains of life and composed of two, three, or six cupredoxin-like domains, oxidize various substrates including transition metals by strict four-electron reduction of dioxygen.^32^ Well-studied and predicted ferroxidases comprise three-domain (3d) MCOs related to Fet3p (Fungi), and 6d-MCOs related to Fox1 (Chlorophyta) or ceruloplasmin and hephaestin (Mammalia).^34^^,^^61^ Guided by the high homology of the LPR1 model with the experimental structure of *Bacillus* CotA laccase, a 3d-MCO, we focused our cladistic analysis on 3d-MCO proteins, the largest group in the MCO superfamily,^32^ and conducted structure-function studies (Figures 1 and S3). Although LPR1 and Fet3p share similar catalytic parameters and an equivalent Fe^2+^-binding and adjoining T1 Cu site,^48^ LPR1 typifies an ancient ferroxidase cohort that emerged early during bacterial land colonization (Figure 7A). The hallmark of LPR1-type ferroxidases, which are only distantly related to yeast ferroxidases and plant laccases (Figure 6E), is a distinctively configured Fe^2+^-binding site that possibly gave rise to the organic substrate-binding pocket in CotA-like MCOs (Figure 6C; Data S3). Gram-positive soil bacteria such as *Bacillus* species produce endospores reinforced with spore-coat proteins to survive in harsh environments.^62^ Although the precise function of CotA is not known, its substrate-binding cavity is unusually large among MCO laccases.^33^

Physical encounters between soil or freshwater bacteria and the terrestrial/subaerial common ancestor of streptophytes likely facilitated HGT of LPR1-type ferroxidases before the divergence of Zygnomatophyceae (or Coleochaetophyceae) and embryophytes (∼580 mya).^55^ Our study supports the recent conjecture that multiple HGT events from soil bacteria to streptophyte progenitors accelerated plant terrestrialization.^50^^,^^55^^,^^63^^,^^64^ For example, gene orthologs of two protein families that enforce land plant resilience, the GIBBERELLIC ACID INSENSITIVE [GAI]/REPRESSOR OF GAI/SCARECROW (GRAS) transcription factors and PYRABACTIN RESISTANCE1 [PYR1]/PYR1-LIKE/REGULATORY COMPONENTS OF ABSCISIC ACID RECEPTORS (PYR/PYL/RCAR)-like abscisic acid receptors, are present in soil bacteria and Zygnomatophyceae algae. Phylogenetic analyses of both gene families indicate HGT from soil bacteria followed by diversification in the embryophytes.^55^ Notably, at least two GRAS members control SCN identity and RAM maintenance in *A. thaliana*,^65^ processes inhibited by LPR1-dependent callose deposition in Pi-deprived root tips.^16^^,^^18^ The Zygnomatophyceae develop rhizoids for substrate adhesion, primordial appendages that evolved to the rhizoid-based rooting systems of bryophytes, and to the rhizomatous (rhizoid-bearing) axes of early vascular plants for anchorage and mineral nutrient scavenging.^52^^,^^66^ Thus, the prospect arises that HGT facilitated the evolution of local Pi sensing circuits in the embryophytes.

The unknown extent of HGT among bacteria obstructs donor identification and possibly explains why LPR1-type MCOs of four phyla (Proteobacteria, Chloroflexi, Actinobacteria, and Firmicutes) are monophyletic with the streptophytes (Figure 6E). However, the metabolic lifestyle of extant bacterial sister genera may allow insight into the function of plant LPR1-like ferroxidases. Members of the four phyla are facultative anaerobic or microaerophilic, spore-forming chemo-organotrophs, which are capable of dissimilatory Fe^3+^ reduction and were isolated from Fe-rich soils or Fe(III) oxide-enriched artificial substrates.^67^^,^^68^ For example, genera of the Geobacteraceae, including *Geobacter* and *Desulfuromonas* species, are the predominant Fe^3+^ reducers in many anaerobic sediments and chemotactically locate Fe(III) minerals for electron transfer via nanowires.^69^ Other Fe-mining strategies involve the release of organic chelating ligands^67^ or soluble electron shuttles,^70^ such as redox-active antibiotics that are controlled by *PHOSPHATE* (*PHO*) regulons and concurrently increase Pi bioavailability.^71^ Intriguingly, anaerobic *Geobacter* species tolerate episodes of dioxygen exposure and encode ROS-scavenging proteins.^72^ Notably, *G. metallireducens* contains four CotA-like genes presumably acquired from *Bacillus*,^73^ two of which encode LPR1-type MCOs (Figure 7A). A fifth gene expresses a 3d-MCO laccase with a very low apparent *K*_M_ for dioxygen (<10 μM).^73^ If low *K*_M_ (O_2_) values are common for MCO enzymes,^74^ bacterial LPR1-type ferroxidases may promote Fe redox cycling to protect against oxidative stress caused by Fe^3+^ reduction and resultant Fe^2+^ Fenton chemistry.

We hypothesize that LPR1-like plant ferroxidases facilitate analogous processes in the apoplast of root tips (Figure 7B). A screen for *lpr1*-like insensitive mutants in *A. thaliana* identified the transcription factor SENSITIVE TO PROTON RHIZOTOXICITY 1 (STOP1) and one of its target, *ALUMINUM-ACTIVATED MALATE TRANSPORTER 1* (*ALMT1*), which is largely expressed in the *LPR1* domain.^15^^,^^21^ In Pi limitation, STOP1-ALMT1 activate malate efflux into the rhizosphere and internal root apoplast^15^ to mobilize by metal ion chelation insoluble Pi from soil minerals^5^^,^^6^ and cell wall-bound Fe–Pi complexes,^4^^,^^7^^,^^15^^,^^16^^,^^21^ respectively. Reduction of Fe^3+^-malate by ascorbate in the apoplast,^75^ possibly expedited by PR upon root illumination,^39^ promotes ROS formation via Fe^2+^ Fenton chemistry, which triggers callose deposition and other cell wall modifications to adjust RAM activity.^15^^,^^16^^,^^21^^,^^24^^,^^31^ Here, we provide evidence for LPR1 function in Fe redox cycling, which is sensitive to Pi-dependent Fe availability and Fe speciation in the root apoplast, as indicated by *in situ* LPR1 activity in root cap cells of transgenic (*CaMV 35S*_*pro*_*:LPR1*) plants (Figures 5 and S5). Because *LPR1* expression and LPR1 abundance are not responsive to Pi or Fe supply (Figure 2), Pi-dependent substrate (Fe^2+^) availability largely determines LPR1 ferroxidase activity and root tip growth (Figures 3 and 4). Our observation that PDR2 opposes LPR1 function by maintaining Fe homeostasis in root meristems supports this proposition (Figure 3C), and it rationalizes the triphasic growth response of Pi-deprived WT roots to increasing Fe supply (Figure 3A). Although root growth inhibition in high Fe (>100 μM) is Pi-independent and caused by Fe toxicity,^36^ the front biphasic segment likely results from PDR2-dependent compensatory processes that are primed in low-Fe condition to counteract excess ROS formation at rising Fe availability, and thus to maintain plasticity of the adaptive response to Pi limitation, a phenomenon known as hormesis.^76^

As studied in yeast and mammalia, coupling of Fe trafficking with ferroxidation minimizes Fe^2+^ escape and Fenton chemistry. Fet3p and hephaestin are tethered to the plasma membrane via a C-terminal transmembrane domain, facilitating direct interaction and Fe channeling between the ferroxidases and Fe permeases.^77^ Although LPR1 is a soluble ferroxidase and physical LPR1 interactors are elusive, the histochemical phenotypes of the *lpr1lpr2* RAM (Figure 5) suggest coupling of LPR1 activity to yet unknown Fe acquisition processes in root tips, which is further supported by our previously published data.^16^^,^^24^ Intriguingly, despite its small surface area, the root cap contributes substantially to plant Pi acquisition, accounting for about 20% of root-imported Pi.^12^ Thus, ALMT1-promoted Fe^3+^ reduction in the root apoplast, particularly in the SCN-delimited *LPR1* expression domain, together with the proposed coupling of LPR1-dependent Fe^2+^-uptake and ferroxidation, likely generate a local transient cue (i.e., ROS formation) of low external Pi availability (Figure 7B).

The regulatory networks of local Pi sensing in plants are just beginning to emerge. As expected, the STOP1-ALMT1 and LPR1-PDR2 modules are not direct targets of the PHR1/PHL-controlled circuits of systemic Pi signaling.^15^ First evidence implicates CLAVATA3/EMBRYO SURROUNDING REGION14 (CLE14) peptide signaling in LPR1-dependent RAM differentiation on low Pi,^31^ and Fe-modulated brassinosteroid signaling in fine-tuned *LPR1* expression in the root elongation zone.^78^ Several studies point to a coordination of diurnal shoot demand for edaphic resources with metabolic and developmental root adjustments,^79^, ^80^, ^81^, ^82^ such as the control of Pi-guided root growth by blue light signaling.^83^^,^^84^ Blue light perception by the CRYPTOCHROME (CRY1/CRY2) photoreceptors in the shoot and translocation of the stabilized ELONGATED HYPOCOTYL 5 (HY5) transcription factor to the root activate *LPR1*, a direct HY5 target gene, to attenuate root meristem activity on low Pi via Fe- and ROS-dependent callose deposition, which supports a function of LPR1-PDR2 in local Pi sensing.^84^ It has recently been argued that root growth inhibition on low Pi in laboratory settings (transparent growth media) is not a biological response but simply a consequence of artificial root exposure to blue light, which triggers photo-Fenton chemistry.^39^ Based on our results (Figures 5 and S5), we do not necessarily disregard this hypothesis, which is contradicted by Gao et al.^84^ However, we propose that illumination of roots with UV or blue light accelerates ALMT1-initiated Fe^3+^-malate reduction by, e.g., ascorbate. Alternatively, root illumination mimics in a fast-tracked manner (few days after seed germination) the more long-term impact of soil bacterial communities in the rhizosphere (weeks to months), which mobilize redox-active Fe^2+^ from Fe(III) oxide minerals by various metabolic processes (Figure 7B).^67^^,^^70^^,^^71^

In conclusion, *Arabidopsis* LPR1 typifies an ancient, hitherto unrecognized ferroxidase cohort that arose early during bacterial land colonization. A recent study identified two major episodes of HGT events during the evolution of streptophytes, corresponding to the early evolution of streptophyte algae and the origin of embryophytes, respectively.^64^ Phylogenomics indicates the common ancestor of Zygnematophyceae and embryophytes acquired a LPR1-type MCO from soil bacteria during the first episode, corroborating previous notions that overcoming the challenges of plant terrestrialization profoundly benefited from HGT events.^52^^,^^55^ Although a function of LPR1 in root Pi sensing has been established in *Arabidopsis*,^15^^,^^16^^,^^18^^,^^20^^,^^21^^,^^31^ which is likely conserved in the tracheophyte lineage (vascular plants) as recently reported for rice,^46^^,^^47^ the biological role of LPR1-like ferroxidases for Pi nutrition in the bryophyte lineage (nonvascular plants) and in extant Zygnematophyceae algae remains an open question. Elucidation of *LPR1*-like gene function across the land plants will require reverse genetics in a bryophyte system,^85^ and genetically tractable model systems are beginning to emerge in the Zygnematophyceae.^86^^,^^87^ *Arabidopsis* LPR1, the most thoroughly characterized plant ferroxidase, provides an impetus for structural biology and biochemistry to study the evolution of bacterial MCOs and to unravel the metabolic functions of LPR1-type MCO ferroxidases in microbial biogeochemistry. Our study points to a regulatory link between local Pi sensing, LPR1 function, and root development in the context of antagonistic metal-Pi interactions. We propose LPR1-type ferroxidases detect subtle differences in Fe substrate availability as a Pi-dependent cue to adjust root meristem activity to external Pi status via processes most likely initiated by Fe redox cycling. The complex biochemical regulation of Fe homeostasis in the root tip apoplast and its unexplored intersection with microbial soil chemistry in the rhizosphere will be major directions of research. Finally, our observation that PDR2/AtP5A counteracts LPR1 function by maintaining Fe homeostasis in root meristems points to a new role of the enigmatic ER-resident P5A-type ATPases in plants,^28^, ^29^, ^30^^,^^88^ which warrants future investigation.

## STAR★Methods

### Key resources table

**Table:** 

REAGENT or RESOURCE	SOURCE	IDENTIFIER
**Antibodies**		

Rabbit polyclonal anti-LPR1	This Study	N/A
Mouse monoclonal anti-actin	Sigma-Aldrich	Cat# A0480; RRID:AB_476670
anti-His-HRP	Miltenyi Biotec	Cat# 130-092-785; RRID:AB_1103231
Goat monoclonal anti-mouse IgG-HRP	BioRAD	Cat# 170-6516; RRID:AB_11125547
Goat monoclonal anti-rabbit IgG-HRP	Thermo Scientific	Cat# 31460; RRID:AB_228341

**Bacterial strains**		

*E. coli* (DH5α)	NEB	C2987K
*E. coli* (Top10)	N/A	N/A
*E. coli* (ArcticExpress (DE3) RIL)	Agilent	230193
*A. tumefaciens* GV3101	N/A	N/A

**Chemicals, peptides, and recombinant proteins**		

Phytoagar	Duchefa	P1.5000
DL-Phosphinothricin	Sigma-Aldrich	45520
IPTG	Sigma-Aldrich	16758
Ni-Agarose	Biozym	2631103
Imidazole	Sigma-Aldrich	15513
N,N-Dimethylthiourea (DMTU)	Sigma-Aldrich	D188700
Carboxy-H2DCFDA	Invitrogen	Lot1911711
dsDNase	Thermo Scientific	EN0771
λ-protein phosphatase	NEB	P0753S
cOmplete, EDTA-free protease inhibitor cocktail	MERCK	11873580001
3-(2-pyridyl)-5,6-bis(2-[5-furylsulfonic acid])-1,2,4-triazine (ferrozine)	Sigma-Aldrich	82940
2,2’-Azino-bis(3-ethylbenzothiazoline-6-sulfonic acid) (ABTS)	Sigma-Aldrich	10102946001
Laccase	Sigma-Aldrich	38429
Bilirubin oxidase	Sigma-Aldrich	B0390
Ceruloplasmin	Athen Research	16-16-030518

**Critical commercial assays**		

peqGOLD Plant RNA Kit	VWR	13-6627-01
SYBR Green	Applied Biosystems	4385612
Fermentas First Strand cDNA Synthesis Kit	Thermo Scientific	K1612
Quick Change II Site-directed mutagenesis Kit	Agilent	200521
2D-Quant	GE Healthcare	80-6483-56
Protein Deglycosylation Mix II	NEB	P6044S

**Experimental models**		

Arabidopsis thaliana Col-0	NASC	N1092
Arabidopsis thaliana *lpr1 lpr2*	Müller et al.^16^	*lpr1 lpr2*
Arabidopsis thaliana *lpr1*	Svistoonoff et al.^20^	NASC: N516297, SALK_016297
Arabidopsis thaliana *pdr2-1*	Ticconi et al.^18^	*pdr2-1*
Arabidopsis thaliana *LPR1*_*pro*_*:eGFP-GUS*	Müller et al.^16^	*LPR1*_*pro*_*:eGFP-GUS*
Arabidopsis thaliana *LPR1*_*pro*_*:eGFP-GUS in pdr2-1*	This study	*LPR1*_*pro*_*:eGFP-GUS in pdr2-1*
Arabidopsis thaliana *PDR2*_*pro*_*:eGFP-GUS*	Ticconi et al.^18^	*PDR2*_*pro*_*:eGFP-GUS*
Arabidopsis thaliana *35S*_*pro*_*:LPR1*	Müller et al.^16^	*35S*_*pro*_*:LPR1*
Arabidopsis thaliana *35S*_*pro*_*:LPR1* in *lpr1*	This study	*35S*_*pro*_*:LPR1* in *lpr1*
Arabidopsis thaliana *35S*_*pro*_*:LPR1*^*E269A*^ in *lpr1*	This study	*35S*_*pro*_*:LPR1*^*E269A*^ in *lpr1*
Arabidopsis thaliana *35S*_*pro*_*:LPR1*^*D370A*^ in *lpr1*	This study	*35S*_*pro*_*:LPR1*^*D370A*^ in *lpr1*
Arabidopsis thaliana *35S*_*pro*_*:LPR1*^*D462A*^ in *lpr1*	This study	*35S*_*pro*_*:LPR1*^*D462A*^ in *lpr1*

**Software and algorithms**		

Proteome Discoverer Version 2.3.	Thermo Scientific	https://thermo.flexnetoperations.com/control/thmo/login
ImageJ (Fiji)	NIH (Public Domain)	https://imagej.nih.gov/ij/
Zen Blue lite edition Version 6.2.9200.0	Zeiss	https://www.zeiss.de/mikroskopie/produkte/mikroskopsoftware/zen-lite.html
MS Amanda Version 2.3.0.14114	Dorfer et al.^89^	https://www.pd-nodes.org/index.php?action=ms-amanda
Mascot Version 2.5.0	Matrix Science	https://www.matrixscience.com/
MOE Version 2020.09	Chemical Computing Group Inc.	https://www.chemcomp.com/Products.htm

### Resource availability

#### Lead contact

Further information and requests for resources and reagents should be directed to and will be fulfilled by the lead contact, Steffen Abel (steffen.abel@ipb.-halle.de).

#### Materials availability

Plant lines and plasmids used in this study will be made available upon request without any restriction.

### Experimental model and subject details

#### Plant material

*Arabidopsis thaliana* accession Columbia (Col-0), Col-0 mutant lines *pdr2-1*, *lpr1lpr2*, and transgenic lines *CaMV 35S*_*pro*_*:LPR1* (OxL1) and *LPR1*_*pro*_*:eGFP-GUS* were previously described.^16^^,^^18^^,^^20^ GATEWAY technology (Invitrogen) and *Agrobacterium*-mediated transformation were used to generate transgenic *Arabidopsis* lines (*lpr1*) expressing *CaMV 35S*_*pro*_*:LPR1*^*E269A*^*, CaMV 35S*_*pro*_*:LPR1*^*D370A*^ and *CaMV 35S*_*pro*_*:LPR1*^*D462A*^. Prior to plant transformation, the integrity of all plasmids was confirmed by DNA sequencing. Transformants (T1 plants) were selected on culture media containing 100 μM DL-phosphinothricin. All experiments were carried out using T4 plants harboring a single T-DNA insert.

#### Plant growth conditions

Seeds were surface-sterilized by exposure (45 min) to chlorine gas (generated in a 5-L desiccator by the addition of 5 ml fuming HCl to 10 ml of 12% [w/v] NaClO), followed by recovery in air for 3 h. After stratification in the dark (2 d at 4 °C), sterilized seeds were germinated on 1% (w/v) Phyto-Agar (Duchefa) containing 2.5 mM KH_2_PO_4_, pH 5.6 (high Pi or +Pi medium) or no Pi supplement (low Pi or –Pi medium), 50 μM Fe^3+^-EDTA, 5 mM KNO_3_, 2 mM MgSO_4_, 2 mM Ca(NO_3_)_2_, 2.5 mM MES-KOH, pH 5.6, 70 μM H_3_BO_3_, 14 μM MnCl_2_, 10 μM NaCl, 0.5 μM CuSO_4_, 1 μM ZnSO_4_, 0.2 μM Na_2_MoO_4_, 0.01 μM CoCl_2_ and 5 g/l sucrose. The agar was routinely purified by repeated washing in deionized water and subsequent dialysis using DOWEX G-55 anion exchanger.^18^ ICP-MS analysis of the washed agar (7.3 μg/g Fe and 5.9 μg/g P) indicated a contribution of 1.3 μM Fe and 1.9 μM P to the solid 1% agar medium. If not stated otherwise, seedlings were vertically grown on agar medium (translucent square petri dishes) in an environmentally controlled walk-in chamber (Johnson Controls, model JC-ESC 300) at constant temperature (21 ^°^C) and 60% relative humidity under cool-white fluorescent illumination at a photon fluence rate of approximately 180 μmol m^-2^ s^-1^ for 16 h daily. In –Pi medium, photoreduction converted approximately 50% of Fe^3+^-EDTA to Fe^2+^ as determined by ferrozine assay (see below). For root length measurements, 27-54 seedlings were transferred to the indicated media and gain of primary root length was marked daily. Photos were analyzed using ImageJ software. Additional lateral roots were induced as previously described.^90^ Hydroponically grown seedlings were germinated under moderate shaking in 200-ml flasks containing 50 ml liquid +Pi medium. We built modified D-Root devices^37^ to allow for light-exposed shoot but light-protected root growth in vertically oriented petri dishes (Figure S5A). For dimethylthiourea (DMTU) treatments, seedlings were germinated on +Pi medium (25 μM Fe) and transferred to agar media containing 750 μM DMTU (solubilized in ddH_2_O).

### Method details

#### Microscopy

Green fluorescent protein (GFP) fluorescence was visualized using a Zeiss LSM 780 confocal laser-scanning microscope (excitation 488 nm, emission 536 nm) in phosphate-buffered saline. Colocalization of GFP and PI (propidium iodide) was monitored in sequential mode (excitation 561 nm, emission 630 nm). Seedlings were incubated for 2 min in 0.1 mg/ml PI solution. For GUS (β-glucuronidase) staining, seedlings were incubated in 50 mM Na-phosphate (pH 7.2), 0.5 mM K_3_Fe(CN)_6_, 0.5 mM K_4_Fe(CN)_6_, 2 mM X-Gluc, 10 mM EDTA, and 0.1% (v/v) Triton X-100 at 37°C and subsequently cleared using chloral hydrate solution (7:7:1, chloral hydrate:ddH_2_O: glycerol) as described before.^91^ Callose was stained for 1 h with 0.1% (w/v) aniline blue (AppliChem) in 100 mM Na-phosphate buffer (pH 7.2) and carefully washed twice. Fluorescence was visualized using a Zeiss LSM 880 confocal laser-scanning microscope (excitation 405 nm, emission 498 nm) in 100 mM Na-phosphate buffer (pH 7.2).^16^ Histochemical iron detection, based on Perls staining coupled to diaminobenzidine (DAB) intensification (Perls/DAB staining) was performed as previously described^16^ with minor changes to the protocol. Plants were incubated for 10 min in 2% (v/v) HCl, 4% (w/v) K-ferrocyanide (Perls staining), or K-ferricyanide (Turnbull staining). For DAB intensification, plants were washed twice (ddH_2_O) and incubated (15 min) in methanol containing 10 mM Na-azide and 0.3% (v/v) H_2_O_2_. After washing with 100 mM Na-phosphate buffer (pH 7.4), plants were incubated for 3 min in the same buffer containing 0.025% (w/v) DAB (Sigma-Aldrich) and 0.005% (v/v) H_2_O_2_. The reaction was stopped by washing with 100 mM Na-phosphate buffer (pH 7.4) and optically clearing with chloral hydrate, 1 g/ml 15% (v/v) glycerol. Production of ROS was measured with the fluorogenic dye 2′,7′-dichlorodihydrofluorescein diacetate (H_2_DCFDA), a cell-permeant compound, as previously described.^16^ Seedlings were incubated in 100 mM Na-phosphate (pH 7.2) supplemented with 10 μM Carboxy-H_2_DCFDA (Invitrogen) for 15 min and subsequently imaged in 100 μM Na-phosphate (pH 7.2).

#### Real-time quantitative PCR

Total RNA was prepared from excised root tips (tip growth gained after seedling transfer) by using the peqGOLD Plant RNA Kit (VWR). One biological replicate represents 40-60 pooled root tips. RNA samples were dsDNase treated (dsDNase, Thermo Scientific, EN0771) and quantified. cDNA was prepared using 1 μg total RNA, which was reverse transcribed by using oligo(dT)_18_ with a Thermo Scientific Fermentas First Strand cDNA Synthesis Kit according to the manufacturer’s protocol. Quantitative real-time PCR was performed in a total volume of 10 μL, containing 1 μL translated cDNA, 5 μL 2x Fast SYBR Green Mix, and 0.75 μM of forward and reverse amplimers, in a Quant Studio 5 System (Applied Biosystems) using the fast run mode. The following cycling program was used: initial denaturation at 95 °C (20 sec), 40 cycles at 95 °C (10 sec) / 60 °C (20 sec), followed by melt curve analysis to confirm the absence of off-target amplification. Gene expression values were calculated by the ΔCt-method using the endogenous *UBC9* (ubiquitin-conjugating enzyme 9) gene as reference gene. All amplimers used for RT-qPCR are listed in Data S6.

#### Quantitative proteomics

Primary root tips were excised and stored in liquid nitrogen. Tissue lysis, sample preparation, protein labeling, Tandem-Mass-Tag (TMT) spectrometry, MS/MS data analysis, and TMT-quantifications were performed as recently described.^92^^,^^93^ MS/MS Data analysis: Raw files were processed with Proteome Discoverer (version 2.3, Thermo Fisher Scientific, Bremen, Germany). Database searches were performed using MS Amanda (version 2.3.0.14114) against the TAIR10 database (32,785 sequences). The raw files were loaded as fractions into the processing workflow. Carbamidomethylation of cysteine and TMT on peptide N-termini were specified as fixed modifications, phosphorylation on serine, threonine, and tyrosine, oxidation of methionine, deamidation of asparagine and glutamine, TMT on lysine, carbamylation on peptide N-termini, and acetylation on protein N-termini were set as dynamic modifications. Trypsin was defined as the proteolytic enzyme, cleaving after lysine or arginine. Up to two missed cleavages were allowed. Precursor and fragment ion tolerance were set to 5 ppm and 15 ppm, respectively. Identified spectra were rescored using Percolator, and filtered to 0.5% FDR at the peptide spectrum match level. Protein grouping was performed in Proteome Discoverer applying strict parsimony principle. Proteins were subsequently filtered to a false discovery rate of 1% at protein level. Phosphorylation sites were localized using IMP-ptmRS implemented in Proteome Discoverer using a probability cut-off of >75% for unambiguous site localization. TMT-quantification: TMT reporter ion S/N values were extracted from the most confident centroid mass within an integration tolerance of 20 ppm. PSMs with average TMT reporter S/N values below 10 as well as PSMs showing more than 50% co-isolation were removed. Protein quantification was determined based on unique peptides only. Samples were sum normalized and missing values were imputed by the 5% quantile of the reporter intensity in the respective sample. Statistical significance of differentially abundant proteins was determined using limma statistical tests.

#### LPR1 homology modeling

YASARA 13.9^94^^,^^95^ was used to derive 25 homology models of LPR1 or bacterial MCOs, each based on five PDB (The Protein Data Bank)^96^ templates (five X-ray structures of laccase CotA from *B. subtilis*:PDB: 2WSD, PDB: 2X88,PDB: 4AKO,PDB: 2X87, and PDB: 4AKP). Quality analysis with PROCHECK^97^ and PROSA II^98^^,^^99^ identified the best fit for each protein. All Cu^+^ cations of the templates were adopted and merged into the models. The ferrous iron (Fe^2+^) was manually added in proximity to residues E269 and D370 of the highly conserved acidic triad on LPR1 or the respective conserved positions in the bacterial ferroxidase models. Subsequently, the model was refined by 20 cycles of simulated annealing refinement with the corresponding tool of YASARA. Molecular surfaces were created with the modeling program MOE (Molecular Operating Environment v2019.0101, Chemical Computing Group Inc., Montreal, QC, Canada, 2019). For determination of root mean square deviation (RMSD) of atomic positions between protein models, amino acid sequences were first pairwise aligned and the corresponding Cα backbone atoms were subsequently superimposed using the MOE program (version 2020.09). Graphical representation of plotted RMSD values (Å) comparing all corresponding amino acid residues are shown in Figure S7D.

#### LPR1 site-directed mutagenesis

To introduce point mutations into plasmid-borne cDNA encoding LPR1 (At1g23010) variants, site-directed mutagenesis was carried out with the Quick Change II Site-directed mutagenesis Kit (Agilent) according to the manufacturer’s instructions. Briefly, two complementary primers containing the desired mutation of the plasmid were used to amplify two overlapping, complementary strands of the plasmid with staggered nicks. After amplification, the parental DNA was digested with *Dpn* I, and the mutated plasmids were transformed into *E. coli* Top 10 or XL1 Blue cells. All bacterial strains were confirmed by plasmid DNA sequencing. See Data S6 for mutagenic oligomers.

#### Purification of native LPR1 protein variants

Transgenic *A. thaliana lpr1* mutant plants expressing *CaMV 35S*_*pro*_*:LPR1*, *CaMV 35S*_*pro*_*:LPR1*^*E269A*^*, CaMV 35S*_*pro*_*:LPR1*^*D370A*^ and *CaMV 35S*_*pro*_*:LPR1*^*D462A*^ were grown for 8 weeks on soil in short-day conditions (8 h light, 16 h darkness, 21°C). Entire plant rosettes were harvested and homogenized in liquid nitrogen. To extract whole proteins, 15 g plant material was vortexed in 40 ml buffer A (50 mM Tris-Cl pH 6.8, 100 mM NaCl, 0.5 mM EDTA, 10% v/v glycerol) containing 1 mM PMSF and 1× protease inhibitor (ROCHE) followed by incubation for 30 min at 4°C (shaking). After clearance of the extract by centrifugation (500 × *g*, 30 min, 4°C), the supernatant was subjected to 40% saturation (NH_4_)_2_SO_4_ precipitation (1 h at 4°C). The resulting pellet (4,500 × *g*, 45 min, 4°C) was discarded and the supernatant treated with 80% (NH_4_)_2_SO_4_ for 1 h at 4°C. The resulting protein pellet was solubilized in 2-3 ml buffer A, loaded on a HighLoad Superdex 200 gel filtration column (HL 16/60, GE Healthcare), and eluted with buffer A as the mobile phase. Fractions containing LPR1 (detected by immunoblot analysis) were directly applied to cation exchange carboxymethyl-sepharose column (HiTrap CM FF, 1-ml, GE Healthcare) equilibrated with buffer B (20 mM Na_2_HPO_4_-NaH_2_PO_4_, pH 7). Fractions were eluted using a linear salt gradient (0-1 M NaCl) in buffer B. LPR1 eluted at 350 mM NaCl. LPR1-containing fractions were stored at -20^o^C until further use. Purified LPR1 enzyme was stable for up two weeks at -20 ^o^C. LPR1 abundance and activity were confirmed by immunoblot analysis and ferroxidase assays, respectively. Silver-staining was performed on gels that were incubated twice for 20 min each or overnight in fixing solution (10% v/v acetic acid, 40% v/v methanol). Subsequently, the gels were incubated in 30% (v/v) methanol, 1.2 mM NaS_2_O_3_, 829 mM Na-acetate for 30 min, followed by three washing steps (5 min each) in distilled H_2_O. Silver-staining was performed by incubating the gels in an aqueous AgNO_3_ solution (2 mg/ml) for 20 min followed by two washing steps with water. The gels were developed (staining of protein bands) in 236 mM NaCO_3_ containing 0.04% (v/v) formaldehyde, and the reaction was stopped by incubation in 40 mM Na-EDTA.

#### Deglycosylation and phosphatase treatments

Purified LPR1 protein was analyzed using Protein Deglycosylation Mix II (New England Biolabs) according to the manufacturer’s instructions. Fetuin was used as a control. Phosphorylation of purified LPR1 protein was tested according to^100^ using λ-protein phosphatase (New England Biolabs). In brief, root material was harvested in phosphatase buffer supplemented with 1 × protease inhibitor (ROCHE). After addition of 1,200 U phosphatase, reactions were carried out for 90 min at room temperature. Samples were inactivated at 95°C for 5 min and analyzed by immunoblotting.

#### Peptide sequencing

Proteins were digested in-gel with trypsin and further processed as previously described.^101^ Dried peptides were dissolved (5% v/v acetonitrile/0.1% v/v trifluoric acid), injected into an EASY-nLC 1000 liquid chromatography system (Thermo Fisher Scientific), and separated by reverse-phase (C18) chromatography. Eluted peptides were electro-sprayed on-line into a QExactive Plus mass spectrometer (Thermo Fisher Scientific). A full MS survey scan was carried out with chromatographic peak width. MS/MS peptide sequencing was performed using a Top10 DDA scan strategy with HCD fragmentation. MS scans with mass-to-charge ratios (m/z) between 400 and 1300 and MS/MS scans were acquired. Peptides and proteins were identified using the Mascot software v2.5.0 (Matrix Science) linked to Proteome Discoverer v 2.1 (Thermo Fisher Scientific). A precursor ion mass error of 5 ppm and a fragment ion mass error of 0.02 Da were tolerated in searches of the TAIR10 database amended with common contaminants. Carbamidomethylation of cysteine was set as fixed modification and oxidation of methionine was tolerated as a variable modification. Peptide spectrum matches (PSM), the peptide and protein level false discovery rate (FDR) was calculated for all annotated PSMs, peptide groups and proteins based on the target-decoy database model and the percolator module. PSMs, peptide groups and proteins with q-values beneath the significance threshold α=0.01 were considered identified.

#### Immunoblot analysis

Polyclonal LPR1 epitope-specific antibodies were raised in rabbits against a mixture of two synthetic peptides (peptide I: 175-PKWTKTTLHYENKQQ-189; peptide II: 222-VESPFQLPTGDEF-234) and affinity-purified (EUROGENTEC, Seraing, Belgium). Total proteins were extracted from frozen plant material in buffer A (50 mM Tris-HCl, pH 6.8, 100 mM NaCl, 0.5 mM EDTA, 10% v/v glycerol) containing 1 × protease inhibitor (ROCHE). After centrifugation (20,000 × *g*, 10 min, 4 °C), the protein concentration of the supernatant was determined (2D-Quant, GE Healthcare), and proteins were separated by SDS/PAGE on 8-10% (w/v polyacrylamide) gels and transferred to PVDF membranes (Semi-Dry-Blot, GE Healthcare). After transfer, membranes were exposed to blocking buffer (1× TBS, 0.05% w/v Tween, 3% w/v milk powder) at room temperature for 1 h or overnight. To detect LPR1, affinity-purified, peptide-specific anti-LPR1 antibody was used 1:1000 in blocking buffer for 1 h at room temperature or at 4 °C overnight. Horseradish peroxidase-conjugated goat anti-rabbit IgG (BioRad, 1:5000) was chosen as a secondary antibody, and the ECL Select or Prime Western Blotting Detection Reagent (Thermo Fisher) was used for visualization. The epitope-specific anti-LPR1 antibody detects 100 ng purified, native LPR1 protein and recognizes only one major protein of ca. 70 kDa in extracts of the *CaMV 35S*_*pro*_*:LPR1* overexpression line (Figure S1A). Plant specific actin-antibody (Sigma-Aldrich) was used (at a dilution of 1:2,000 in blocking buffer for 1 h at room temperature) as loading control. Horseradish peroxidase-conjugated goat anti-mouse IgG (BioRAD, 1:5000) was chosen as a secondary antibody, and the ECL Select Western Blotting Detection Reagent (Thermo Fisher) was used for visualization. To control for recombinant bacterial multicopper oxidase expression anti-His-HRP (Miltenyi Biotec) was used (at a dilution of 1:10,000) in the blocking buffer.

#### Ferroxidase and other MCO assays

Protein concentration was determined using the Qubit Fluorometric Quantification System (Thermo Fisher) according to the manufacturer’s instructions. All reagents except human ceruloplasmin (Athens Research) were purchased from Sigma-Aldrich. Ferroxidase activity was determined as previously described^16^ using typically 25 μM Fe(NH_4_)_2_(SO_4_)_2_ × 6 H_2_O as the substrate and 3-(2-pyridyl)-5,6-bis(2-[5-furylsulfonic acid])-1,2,4-triazine (ferrozine) as a specific Fe^2+^ chelator to scavenge the remaining substrate after the reactions. The rate of Fe^2+^ oxidation was calculated from the decreased absorbance at 560 nm using a molar extinction coefficient of ε_560_=25,400 M^-1^ cm^-1^ for the Fe^2+^-ferrozine complex.^102^ Ferroxidase kinetic parameters were obtained in triplicate per LPR1 protein preparation using 1 μg pure LPR1 enzyme and different substrate (Fe^2+^) concentrations, ranging from 0 μM to 200 μM Fe(NH_4_)_2_(SO_4_)_2_ × 6 H_2_O. Kinetic data derived from four independent LPR1 preparations (12 data sets) were analyzed using Fig.P Version 2006 (Fig.P Software Incorporated, Hamilton On, Canada) according to the Michaelis-Menten equation (V_max_^∗^[S]) / (*K*_M_ + [S]).^103^ Characterization of LPR1 variants expressed in transiently transgenic tobacco leaves was performed equally using cleared leaf protein extracts. In brief, 4 days after infiltration leaf discs were collected and total proteins were extracted from frozen plant material in buffer A (50 mM Tris-HCl, pH 6.8, 100 mM NaCl, 0.5 mM EDTA, 10% v/v glycerol) containing 1 × protease inhibitor (ROCHE). After centrifugation (20,000 × *g*, 10 min, 4 °C), the protein concentration of the supernatant was determined (2D-Quant, GE Healthcare). Assays included protein extract prepared from untransformed leaves as negative control, which was subtracted as background activity from leaf extracts expressing LPR1 variants. Phenol oxidase (laccase) activity with ABTS (2,2′-azino-bis[3-ethylbenzothiazoline-6-sulfonic acid]), ascorbate oxidase activity, and bilirubin activity were measured in 0.1 M NaH_2_PO_4_-Na_2_HPO_4_ (pH 5.6 – 7.2) as described.^104^, ^105^, ^106^

#### Transient expression assays

The transient transformation of *Nicotiana benthamiana* leaves was carried out using *Agrobacterium tumefaciens* strains that carried the indicated plasmids and the pCB301-p19 helper plasmid.^107^ Bacteria were grown overnight to an OD_600 nm_ = 0.5 – 0.8, harvested (10,000 × *g*, 4 min, 4 °C) and washed two times with 2 ml of transformation buffer (10 mM MES-KOH, pH 5.5, 10 mM MgCl_2_, 150 μg/ml acetosyringone) and subsequently dissolved in transformation buffer to an OD_600_ of 1. The bacteria carrying the expression construct were mixed 1:1 with the ones harboring the pCB301-p19 plasmid and incubated for 1 h at 20 °C. Subsequently, the bacteria were infiltrated at the bottom side of leaves of 5-7 week-old plants, germinated and cultivated in greenhouse conditions (21 °C). Samples were harvested 4 d post infiltration.

#### Expression of bacterial MCO proteins

Genes encoding potential bacterial ferroxidases from *Sulfurifustis variabilis* (GenBank: BAU47383.1) and *Streptomyces clavuligerus* (GenBank: QCS10718.1) were codon-optimized, synthesized at the Invitrogen GeneArt Gene Synthesis platform, and cloned into pVp16-Dest vector for IPTG (isopropyl-β-thiogalactopyranoside)-induced expression (3 h at 37°C) in *E. coli* strain ArcticExpress (Agilent). After sonication, the clarified cell lysates were directly used for ferroxidase activity assays.

#### Phylogenetic analyses

MCO sequence alignment and phylogenetic analysis were done using 187 referenced protein sequences of the annotated ‘multicopper oxidase family’ obtained from uniProt Knowledgebase (www.uniprot.org) and filtered for fragments. CotA (P07788) was added to the dataset. All phylogenetic trees were calculated by sequence alignment using MAFFT 7^108^ with default settings and created at the CIPRES web-portal with RAxML 8.2.10^109^ for maximum likelihood analyses using the JTT PAM matrix for amino acid substitutions in RAxML. All LPR1-related sequences used for phylogenetic analyses are provided (Data S4 and S5).

#### Data base searches

For select land plant species, full-length MCO amino acid sequences similar to *Arabidopsis* LPR1 (At1g23010) were obtained by tblastn searches (filter: >60% query coverage; *e*-value of 1e^-40^ cutoff; >30% identity) of the nonredundant nucleotide collection maintained at NCBI. All sequence hits, mostly annotated as ‘multicopper oxidase LPR1- or LPR2-like or homologs’, were considered for further analysis, irrespective of the presence of a conserved LPR1-type acidic triad (Data S2A)^110^. Sequence hits of the annotated major MCO classes, laccases, bilirubin oxidases and ascorbate oxidases, scored consistently below the filter thresholds (i.e., <50% query coverage; cutoff: *e*-value of 6e^-07^; <30% identity). Representative genomes of the key taxonomic groups of the Bacteria and Archaea were retrieved from NCBI assembly. Transcriptome data of the One Thousand Plant Transcriptomes Initiative,^59^ and the recently released genome data of *Penium magaritaceum*, *Chara braunii*, *Mesostigma viride*, *Chlorokybus sp.*, *Mesotaenium endlicheranum*, *Spiroglea musicola*, and *Klebsormidium* nitens^54^^,^^55^, ^56^, ^57^, ^58^ were included. Blastp (version 2.10.1+)^111^ and hmmer (version 3.3)^112^ were used (www.hmmer.org)^111^^,^^113^ to identify *LPR1*-like genes in the main taxonomic groups of Bacteria, Archaea, algae (glaucophytes, rhodophytes, green algae), and bryophytes. First, the LPR1 (At1g23010) amino acid sequence was used as a query to interrogate each genome or transcriptome. Only hits with an alignment length of >175 amino acid residues (>30% query coverage) were considered. Second, a hidden Markov model profile approach was applied, generating an HMM profile for LPR1-like protein sequences from 14 vascular plant species for scanning each genome or transcriptome. All sequence hits were scanned for the presence of the LPR1-type Fe^2+^-binding site, which is composed of an acidic triad (residues underlined) embedded in three consensus motifs: 1. [WVI]XP[EA][YAF]X[GA]; 2. N[DTS][AG]XXP[YF]PXG[DE]X(5-10)[VI][ML]XF; and 3. NXTX[DEG]XHP. Sequences that cover at least two of the three consensus sequence motifs were considered. Third, for final validation, candidate sequences were aligned with *Arabidopsis* LPR1 and visually inspected for the presence of acidic triad motifs, and for the MCO hallmarks (T1 Cu site; T2/T3 Cu cluster). Lastly, representative contiguous sequences covering all acid triad signature motifs (e.g., aa 264-465 for AtLPR1 or aa 222-420 for CotA) were used as query to interrogate (tblastn searches at NCBI) each bacterial phylum for LPR1-type sequences with incomplete acid triad signatures. For quality control (scaffold anchorage), blast analysis of genes neighboring the *LPR1*-like locus in the bryophyte, *Marchantia polymorpha*, and in select Zygnematophyceae algae, *M. endlicherianum*, *S. musicola*, and *P. magaritaceum*, revealed hits for numerous plant-related genes and for only some bacterial genes, thus excluding contamination. However, no such hits were found in *K.* nitens, questioning the authenticity of the putative *LPR1*-like gene. Analysis of the *LPR1*-like genomic neighborhood in select vascular plants revealed syntenic genes encoding heavy metal associated proteins (HIPPs) in *A.thaliana* (At1g22990; At1g23000), *B. rapa* (LOC103840804; LOC103840806), *V. vinifera* (VIT_00011777001), *C. rubella* (CARUB_v10012305mg; CARUB_v10010550mg) and *S. lycopersicon* (LOC101258259). All LPR1-related sequences used and genomes interrogated in this study are listed (Data S2C and S4).

### Quantification and statistical analysis

Most of the experiments were performed in triplicates or more with number of events measured indicated in the figure legends. Statistical differences were assessed by Student’s *t*-test; one-way or two-way ANOVA and Tukey’s (Honestly Significant Difference) HSD posthoc test, using built-in functions of the statistical environment R (R Development Core Team, 2018). Different letters in graphs denote statistical differences at P < 0.05. Graphs were generated using the ggplot2 R package.

## Data Availability

• Microscopy data reported in this paper will be shared by the lead contact upon request. • No code was generated in this study. • Any additional information required to reanalyze the data reported in this paper is available from the lead contact upon request.

## Supplemental information

Document S1. Figures S1–S7

Data S1. Purification of native *Arabidopsis* LPR1, related to STAR Methods and Figure 1 Native, untagged LPR1 protein was purified from leaves of transgenic *CaMV 35S*_*pro*_*:LPR1* plants (*A. thaliana*). All fractions were assayed for ferroxidase activity at pH 5.8 as described. The average yield of purified LPR1 was approximately 2 μg protein per gram leaf material.

Data S2. Identification of LPR1-type MCO ferroxidases, related to Figures 6 and 7 (A) Distribution of LPR1-type MCO ferroxidases across the domains of life. LPR1-like sequences in the embryophytes were identified by tblastn searches at NCBI using *Arabidopsis thaliana* LPR1 (At1g23010) as the query (see STAR Methods). For the select land plant species, all sequence hits are listed, irrespective of the presence of a conserved LPR1-type acidic triad (E269-D370-D462). An HMM profile of LPR1 (14 vascular plant species) was used to interrogate at NCBI all other domains of life and phyla (see STAR Methods). Sequence hits were filtered and visually inspected to validate the presence of conserved acidic triad motifs necessary for Fe^2+^-binding by LPR1 (see sheet B; Data S3). The HHM profile was used to search transcript data of the One Thousand Plant Transcriptomes (1KP) Initiative,^48^ and the recently published genomes of *Anthoceros angustus*,^47^*Mesotaenium endlicherianum* and *Spirogloea musicola*,^57^*Penium magaritaceum*,^58^*Mesostigma viride* and *Chlorokybus atmophyticus*,^59^ and *Klebsormidium nitens*^46^ (see sheet C). Only sequence hits with a complete LPR1-type acidic triad are listed for the bacterial phyla. Additional bacterial sequences with an incomplete acidic triad are provided in Data S4 (see sheet C; Figure 7A; Data S3). N. d. not detected; [+], motif present; [-], motif absent; [?] acidic residue is present but, at nonconserved position. (B) HMM profile search for sequences related to LPR1-type MCO ferroxidases. An HMM profile of LPR1 (14 vascular plant species) was used to search the domains Bacteria and Archaea (NCBI), and the transcriptome data of Archaeplastida (1KP Initiative), including green plants, glaucophytes and rhodophytes.^48^ Sequence hits were filtered and visually inspected to validate the presence of conserved motifs embedding the acidic triad, E269, D370 and D462 (abbreviated as 1-1-1), necessary for Fe^2+^-binding by *Arabidopsis* LPR1 (see STAR Methods; sheet A; Data S3). Additional searches at NCBI were conducted, using internal polypeptides covering the entire acidic triad segment of *Arabidopsis* LPR1 (aa 264-465) and the corresponding segment in *Bacillus* CotA (aa 222-420) as queries, to identify additional LPR1-related MCO sequences (see sheet C; Data S4) with incomplete, degenerate acid triad motifs (permutations abbreviated as 1-0-1 to 0-0-0). For the 1 KP Initiative data (Archaeplastida), the column ‘No. of species’ gives the number of retrieved species in our study versus the number of species (in parenthesis) reported in by One Thousand Plant Transcriptomes Initiative.^48^ The validated hits (1-1-1) for streptophyte algae also include the genome searches of *Mesotaenium endlicheranum*,^57^*Spiroglea musicola*,^57^*Penium margaritaceum*,^58^ and *Klebsormidium* nitens.^46^ (C) List of genomes scanned for LPR1-like proteins.

Data S3. Alignment of LPR1-related acidic triad motif polypeptide sequences, related to Figures 6 and 7 Exhaustive searches were conducted (NCBI; published genomes;^46,47,57–60^ One Thousand Plant Transcriptomes Initiative^48^) to identify polypeptide sequences related to *Arabidopsis* LPR1 (see STAR Methods; Data S2A, S2C, and S4). Aligned are the conserved motifs embedding the acidic triad of LPR1 relative to E269, D370, and D462 (highlighted in red). Hydrophobic residues are highlighted in grey. Proline residues of the first motif (E269), except at the start, and in the center of the variable linker (flanking D370) are highlighted in petrol blue. Glycine residues in the variable linker segment, and highly frequent or invariant residues in all three motifs are typed in bold face. Aligned are the LPR1-like motif sequences of select embryophytes (from NCBI), of streptophyte algae (identified in published genomes [highlighted in yellow] or by the 1KP project), and of bacterial phyla (from NCBI). Note, sequences for some streptophyte algae are truncated (xxx). Three sequences are too long for reasonable alignment (^∗^, extra peptide sequences on the right).

Data S4. Amino acid sequences of all LPR1-like proteins used in the phylogenetic analyses, related to Figures 6 and 7

Data S5. Alignment of all LPR1-like protein sequences used in this study, related to Figures 6 and 7

Data S6. List of mutagenic oligomers and qRT-PCR amplimers, related to STAR Methods

Document S2. Article plus supplemental information
