# Supplementary material for: Bacterial-type ferroxidase tunes iron-dependent phosphate sensing during Arabidopsis root development
Source: Curr Biol. 2022 May 23;32(10):2189–2205.e6. doi: 10.1016/j.cub.2022.04.005 (PMC9168544; doi:10.1016/j.cub.2022.04.005)
Supplement: Document S1. Figures S1–S7 [file mmc1.pdf]

Current Biology, Volume *32*

Supplemental Information

Bacterial-type ferroxidase tunes iron-dependent phosphate sensing during *Arabidopsis* root development

Christin Naumann, Marcus Heisters, Wolfgang Brandt, Philipp Janitza, Carolin Alfs, Nancy Tang, Alicia Toto Nienguesso, Jörg Ziegler, Richard Imre, Karl Mechtler, Yasin Dagdas, Wolfgang Hoehenwarter, Gary Sawers, Marcel Quint, and Steffen Abel

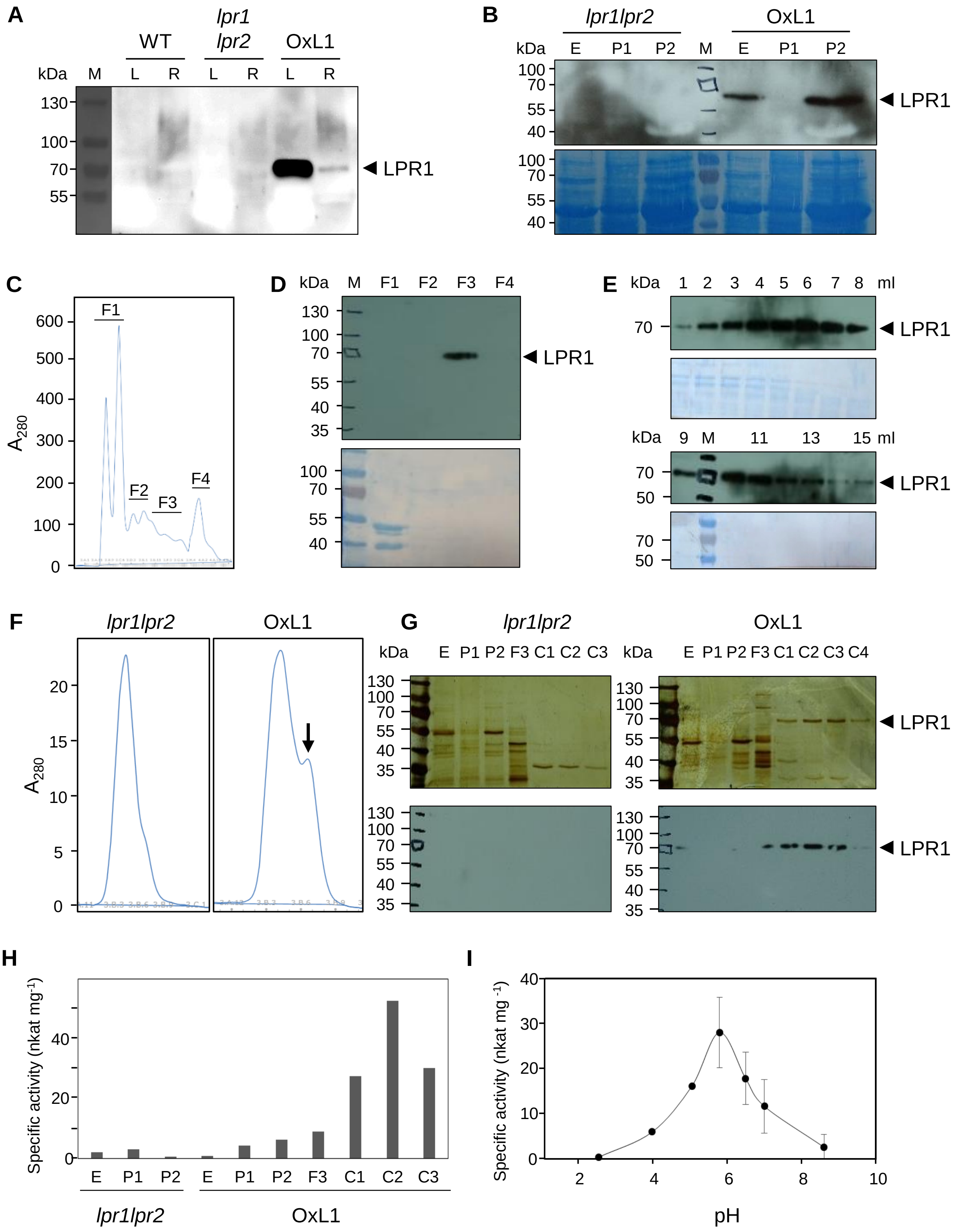

A
WT
lpr1
lpr2
OxL1
kDa
M
L
R
130
100
70
55
LPR1
B
lpr1lpr2
OxL1
E
P1
P2
40
C
F1
F2
F3
F4
600
500
400
300
200
0
A280
D
35
E
1
2
3
4
5
6
7
8
ml
9
11
13
15
50
F
20
15
10
5
G
C1
C2
C3
C4
H
Specific activity (nkat mg-1)
I
pH

Figure S1. Purification of native LPR1 from leaves of LPR1-overexpressing plants, Related to Figure 1

(A) Immunoblot analysis (n>10) of LPR1 expression in leaves (L) and roots (R) of wild-type (WT), *lpr1lpr2*, and transgenic *CaMV* $35S_{pro}$*:LPR1* plants (OxL1). Protein extracts (50 μg protein) of 6-week-old greenhouse-grown (soil) plants were analyzed.
(B) Immunoblot analysis of protein extracts prepared from leaves of 6-week-old greenhouse-grown (soil) *lpr1lpr2* and *CaMV* $35S_{pro}$*:LPR1* plants (OxL1) (lanes E), and of protein pellets of fractions prepared by differential ammonium sulfate precipitation: 40% saturation (lanes P1) and 40-80% saturation (lanes P2). The membrane was stained with Coomassie-Blue.
(C) Elution profile after size-exclusion chromatography. Four major fractions (lanes F1-F4) were pooled for further processing and analysis.
(D) Immunoblot analysis of pooled fractions (lanes F1-F4).
(E) Immunoblot analysis of fraction F3 at 1-ml resolution (1-15 ml).
(F) Elution profiles of protein preparations from *lpr1lpr2* and *CaMV* $35S_{pro}$*:LPR1* (OxL1) plants after cation-exchange chromatography. Note the pronounced shoulder in the OxL1 profile (arrow).
(G) Separation of proteins by SDS-PAGE (upper panels: silver-stained gels) and immunoblot analysis (lower panels) of all relevant fractions prepared from *lpr1lpr2* and *CaMV* $35S_{pro}$*:LPR1* (OxL1) plants.
(H) Specific ferroxidase activities of the indicated fractions.
(I) pH optimum of LPR1 ferroxidase activity in 0.1 M Na-acetate buffer (±SD; n=3).

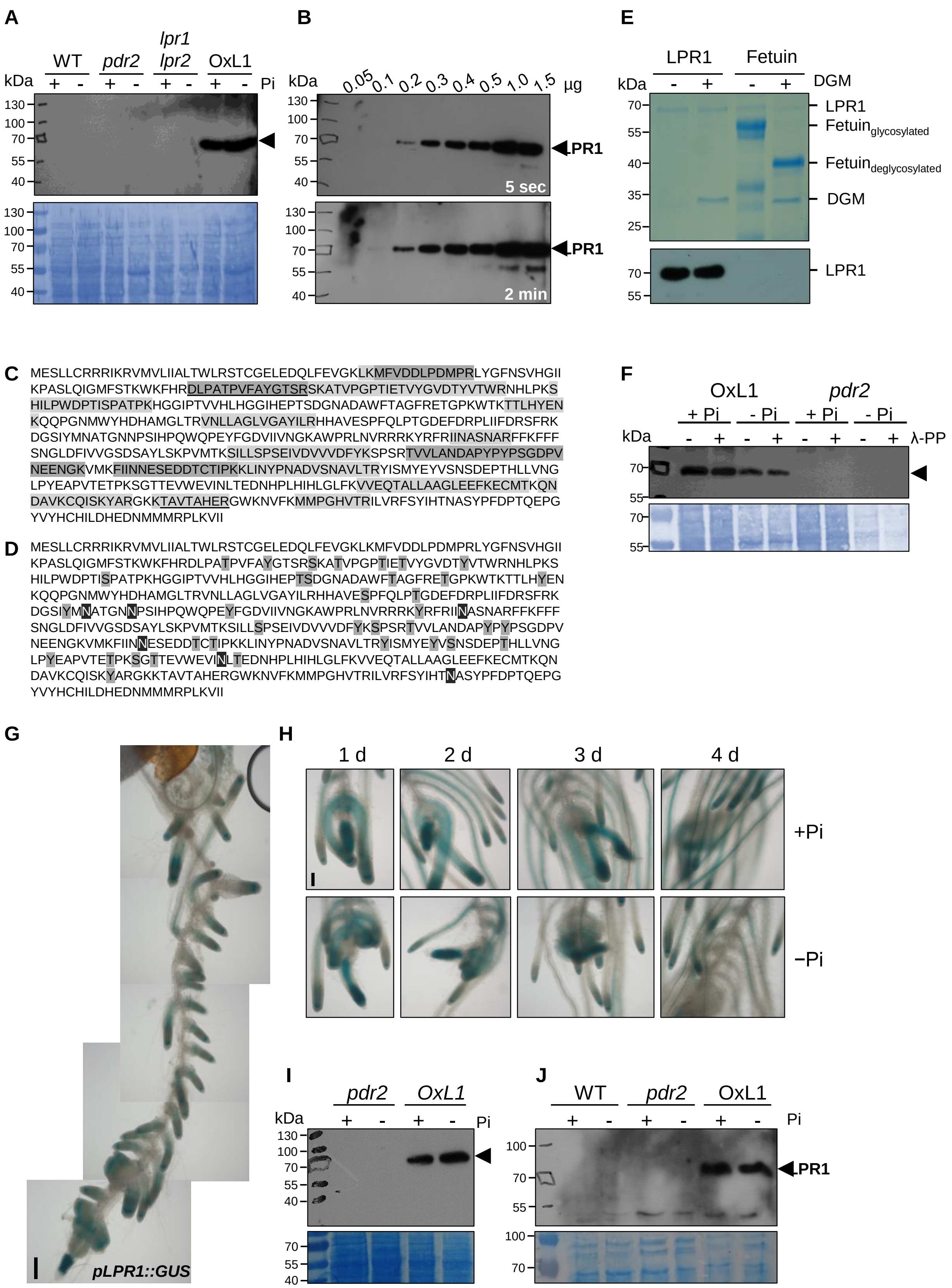

A
WT
pdr2
lpr1 lpr2
OxL1
kDa
+ - + - + - + - Pi
130
100
70
55
40
B
0.05 0.1 0.2 0.3 0.4 0.5 1.0 1.5 µg
LPR1
5 sec
2 min
E
LPR1
Fetuin
DGM
LPR1
Fetuin glycosylated
Fetuin deglycosylated
DGM
C
MESLLCRRRIKRVMVLIIALTWLRSTCGELEDQLFEVGKLKMFVDDLPDMPRLYGFNSVHGII
KPASLQIGMFSTKWKFHRDLPATPVFAYGTSRSKATVPGPTIETVYGVDTYVTWRNHLPKS
HILPWDPTISPATPKHGGIPTVVHLHGGIHEPTSDGNADAWFTAGFRETGPKWTKTTLHYEN
KQQPGNMWYHDHAMGLTRVNLLAGLVGAYILRHHAVESPFQLPTGDEFDRPLIIFDRSFRK
DGSIYMNATGNNPSIHPQWQPEYFGDVIIVNGKAWPRLNVRRRKYRFRIINASNARFFKFFF
SNGLDFIVVGSDSAYLSKPVMTKSILLSPSEIVDVVVDFYKSPSRTVVLANDAPYPYPSGDPV
NEENGKVMKFIINNESEDDTCTIPKKLINYPNADVSNAVLTRYISMYEYVSNSDEPTHLLVNG
LPYEAPVTETPKSGTTEVWEVINLTEDNHPLHIHLGLFKVVEQTALLAAGLEEFKECMTKQN
DAVKCQISKYARGKKTAVTAHERGWKNVFKMMPGHVTRILVRFSYIHTNASYPFDPTQEPG
YVYHCHILDHEDNMMMRPLKVII
D
MESLLCRRRIKRVMVLIIALTWLRSTCGELEDQLFEVGKLKMFVDDLPDMPRLYGFNSVHGII
KPASLQIGMFSTKWKFHRDLPATPVFAYGTSRSKATVPGPTIETVYGVDTYVTWRNHLPKS
HILPWDPTISPATPKHGGIPTVVHLHGGIHEPTSDGNADAWFTAGFRETGPKWTKTTLHYEN
KQQPGNMWYHDHAMGLTRVNLLAGLVGAYILRHHAVESPFQLPTGDEFDRPLIIFDRSFRK
DGSIYMNATGNNPSIHPQWQPEYFGDVIIVNGKAWPRLNVRRRKYRFRIINASNARFFKFFF
SNGLDFIVVGSDSAYLSKPVMTKSILLSPSEIVDVVVDFYKSPSRTVVLANDAPYPYPSGDPV
NEENGKVMKFIINNESEDDTCTIPKKLINYPNADVSNAVLTRYISMYEYVSNSDEPTHLLVNG
LPYEAPVTETPKSGTTEVWEVINLTEDNHPLHIHLGLFKVVEQTALLAAGLEEFKECMTKQN
DAVKCQISKYARGKKTAVTAHERGWKNVFKMMPGHVTRILVRFSYIHTNASYPFDPTQEPG
YVYHCHILDHEDNMMMRPLKVII
F
OxL1
pdr2
+ Pi - Pi + Pi - Pi
kDa
- + - + - + - + λ-PP
G
pLPR1::GUS
H
1 d
2 d
3 d
4 d
+Pi
−Pi
I
pdr2
OxL1
kDa
+ - + - Pi
J
WT
pdr2
OxL1
+ - + - + - Pi
LPR1

Figure S2. Detection, verification, and test for posttranslational modifications of purified native LPR1, Related to Figure 1 and Figure 2

(A) Immunoblot analysis of LPR1 in root extracts of Pi-replete (+Pi) and Pi-deprived (–Pi) wild-type (WT), *pdr2*, *lpr1lpr2*, and *CaMV 35S$_{pro}$:LPR1* (OxL1) plants. Seeds were germinated and vertically grown on +Pi agar medium (5 d) in a controlled environmental chamber at 22°C under cool-white fluorescent illumination at a photon fluence rate of approximately 180 µmol m^{-2} s^{-1} for 16 h daily (16 h L /8 h D). Subsequently, seedlings were transferred to +Pi or –Pi agar medium (vertical translucent square petri dishes) for 1 d (STAR Methods). Root tips (root growth gain) were harvested, protein extracts (104 µg total protein) separated by SDS-PAGE (8% gels), transferred to PVDF membranes, and probed (anti-LPR1). Membranes were stained with Coomassie-Blue (n=3).
(B) Increasing amounts of purified native LPR1 were separated by SDS-PAGE (8% gels), transferred to PVDF membranes and probed (anti-LPR1).
(C) Detection of LPR1-derived peptides. Purified native LPR1 protein was separated by SDS-PAGE and the identity of the eluted protein determined by MS/MS peptide sequencing (n=5). Detected LPR1-derived peptides are highlighted (light grey) on the primary LPR1 structure. Peptides detected in all five measurements are depicted in dark grey. Unique peptides identified in the TMT-dataset (quantitative proteomics) are underlined.
(D) Potential glycosylation (black) and phosphorylation (grey; PhosPhAt 4.0) sites.
(E) Deglycosylation assays with purified LPR1 and fetuin as a positive control. Reactions with or without deglycosylating enzyme mix (DGM) were subjected to SDS-PAGE (10% gels). Proteins were detected by Coomassie-Blue staining or immunoblot analysis (anti-LPR1).
(F) Dephosphorylation assay using root extracts from *CaMV 35S$_{pro}$:LPR1* (OxL1) and *pdr2* plants germinated for 6 d on +Pi or –Pi media with and without Lambda protein phosphatase (n=2). See also (A).
(G) Expression of *LPR1$_{pro}$:GUS* in wild-type plants. Lateral roots were induced (10-15 meristems per mm) by NPA-NAA treatment. Seeds were germinated on +Pi agar medium supplemented with 10 µM NPA for 3 d prior to transfer to +Pi agar supplemented with 10 µM NAA[90]. See also (A). After 4-6 d of transfer, roots were monitored for GUS expression. Shown is one composite representative image (n=11). Scale bar, 100 µm.
(H) Expression of *LPR1$_{pro}$:GUS* in response to Pi. Seeds were germinated for 3 d on +Pi (10 µM NPA), prior to transfer to +Pi (10 µM NAA) to induce lateral roots as above (G). After 4 d of transfer, seedlings were transferred for up to 4 d to +Pi or –Pi agar without supplements and roots were monitored for GUS expression. Shown are representative images (n≥10). Scale bars, 100 µm.
(I) Immunoblot analysis of LPR1 in root extracts prepared from +Pi or –Pi grown *pdr2* and *CaMV 35S$_{pro}$:LPR1* (OxL1) plants. Seeds were germinated in +Pi liquid medium (7 d) prior to the addition of 10 µM NPA (3 d). After media exchange, seedlings were treated with 10 µM NAA (6 d), washed and transferred to liquid +Pi or –Pi media without supplements (3 d). Whole root extracts (51 µg total protein) were blotted and probed (anti-LPR1). Membranes were stained with Coomassie-Blue (n=4).
(J) Immunoblot analysis of LPR1 in fractionated root extracts of +Pi or –Pi grown wild-type (WT), *pdr2*, and *CaMV 35S$_{pro}$:LPR1* (OxL1) plants. See also (A). Seeds were germinated in +Pi (7 d) prior to transfer to +Pi or –Pi media 4 d). Whole root extracts were subjected to ammonium sulfate

precipitation (40% saturation) and supernatants (40 μL) were blotted and probed (anti-LPR1). Membranes were stained with Coomassie-Blue (n=1).

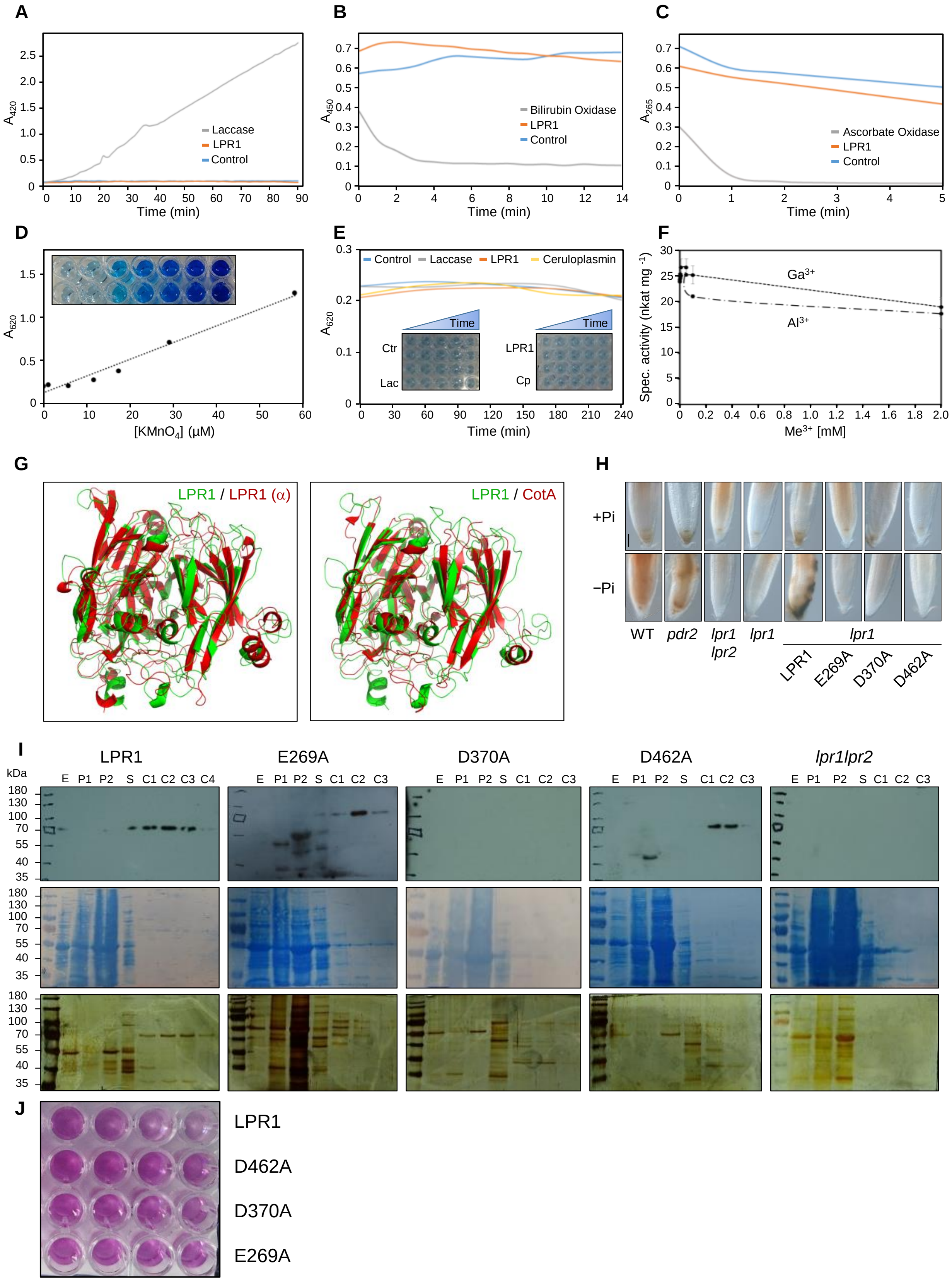
A
B
C
Laccase
LPR1
Control
Time (min)
Bilirubin Oxidase
Ascorbate Oxidase
D
E
F
[KMnO4] (µM)
Ctr
Lac
LPR1
Cp
Time
Ceruloplasmin
Spec. activity (nkat mg -1)
Ga3+
Al3+
Me3+ [mM]
G
LPR1 / LPR1 (α)
LPR1 / CotA
H
+Pi
−Pi
WT
pdr2
lpr1 lpr2
lpr1
E269A
D370A
D462A
I
LPR1
E269A
D370A
D462A
lpr1lpr2
kDa
E
P1
P2
S
C1
C2
C3
C4
J
LPR1
D462A
D370A
E269A

Figure S3. LPR1 substrate specificity and homology modeling, and purification of LPR1 mutant variants, Related to Figure 1

(A) Laccase activity with 0.5 mM ABTS (2,2'-azino-bis[3-ethylbenzothiazoline-6-sulfonic acid]) as substrate and commercial laccase (*Trametes versicolor*) as control (grey, one representative of four assays). LPR1 (orange); no enzyme control (blue).
(B) Bilirubin oxidase activity with 35 µM bilirubin as substrate and commercial bilirubin oxidase (*Myrothecium verrucaria*) as control (grey, one of two assays). LPR1 (orange); no enzyme control (blue) (n =3).
(C) Ascorbate oxidase activity with 60 µM ascorbate as substrate and commercial ascorbate oxidase (*Cucurbita sp.*) as control (grey, one representative of four assays). LPR1 (orange); no enzyme control (blue), (n=3).
(D, E) Test for manganese oxidase activity with 1 mM $MnSO_4$ as substrate (n=3). (D) Calibration curve of $KMnO_4$ (the oxidized product) in the presence of 0.005% (w/v) leucoberbelin blue. (E) Assays with LPR1, laccase (*T. versicolor*) and human ceruloplasmin (ferroxidase), which did not display any detectable manganese oxidase activity.
(F) Test of increasing trivalent metal (Al^{3+} and Ga^{3+}) concentrations on LPR1 ferroxidase activity, (±SD; n=3).
(G) Superposition of LPR1 homology models. The LPR1 homology model (green) generated by YASARA (STAR Methods) was superposed with the LPR1 homology model (red) created by the alphafold (https://alphafold.ebi.ac.uk/entry/F4I4K5) server (left panel), or with the X-ray structure (PDB: 4AKP) of CotA (red) from *B. subtilis* (right panel). The RMSD values for all superposed Cα atoms are 4.0 Å (LPR1/LPR1α) and 3.4 Å (LPR1/CotA), respectively. The variable loop conformations explain the relatively high values.
(H) Site-directed mutagenesis and complementation of *lpr1* plants with LPR1 variants. After germination (5 d, +Pi), seedlings were transferred to +P or –Pi medium (25 µM Fe). Three days after transfer, root tips were monitored for Fe^{3+} accumulation by Perls staining coupled to diaminobenzidine (DAB) intensification. Shown are representative images (n≥15). Scale bars 50µm.
(I) Using the three-step purification protocol (STAR Methods; see Figure S1), untagged LPR1 wild-type or mutant proteins were purified from extracts of transgenic *lpr1* plants expressing under the *CaMV 35S* promoter $LPR1^{WT}$, $LPR1^{E269A}$, $LPR1^{D370A}$ or $LPR1^{D462A}$. Line *lpr1lpr2* was used as negative control. Shown are immunoblots probed with anti-LPR1 (upper row), Coomassie-Blue stained membranes of blots (center row), and silver-stained gels loaded with 1 µg protein per lane prior to separation (lower row). Protein fractions: (lane E) leaf extract; (lane P1) ammonium sulfate (40% saturation) precipitation; (lane P2) ammonium sulfate (40-80% saturation) precipitation; (lane S) size exclusion chromatography; (lanes C1-C4) cation exchange chromatography. (n=3).
(J) Discontinuous ferrozine assay using 1 µg of purified wild-type and mutant LPR proteins.

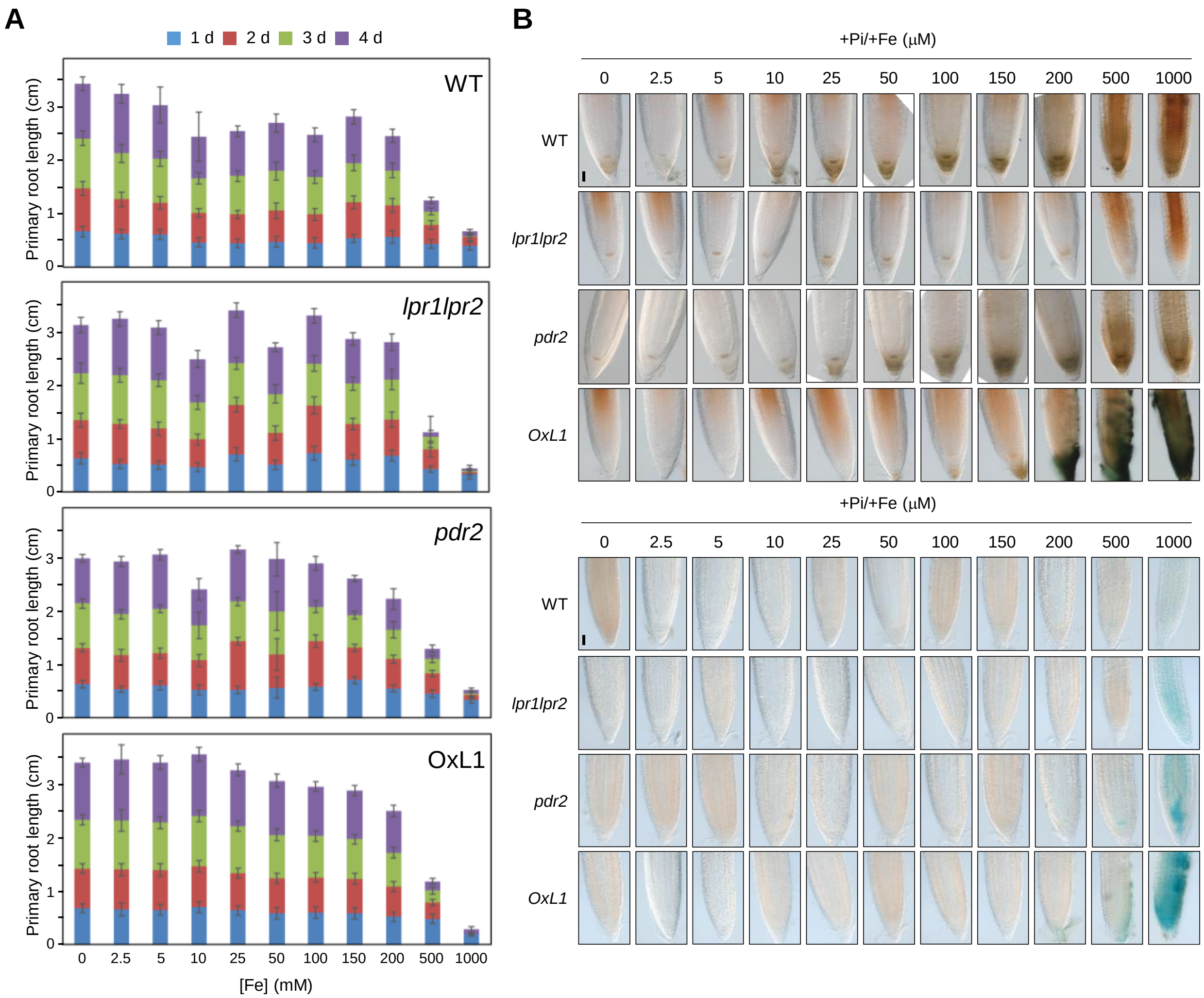
A
1 d 2 d 3 d 4 d
WT
lpr1lpr2
pdr2
OxL1
Primary root length (cm)
0 1 2 3
0 2.5 5 10 25 50 100 150 200 500 1000
[Fe] (mM)
B
+Pi/+Fe (μM)
0 2.5 5 10 25 50 100 150 200 500 1000
WT
lpr1lpr2
pdr2
OxL1
+Pi/+Fe (μM)
0 2.5 5 10 25 50 100 150 200 500 1000
WT
lpr1lpr2
pdr2
OxL1

Figure S4. Fe-dependent primary root extension and Fe^{3+} accumulation in root tips on Pi-replete medium, Related to Figure 3

(A) Iron-dependent primary root extension on Pi-replete medium. Seeds of wild-type (WT), *lpr1lpr2*, *pdr2*, and LPR1-overexpressing plants (OxL1) were germinated on +Pi agar medium (5 d) prior to transfer to +Pi media with increasing iron (Fe^{3+}-EDTA) supply (STAR Methods, see also Figure S2A). Gain of primary root extension was recorded daily for up to 4 d after transfer and plotted for each genotype (±SD; n≥50).
(B) Iron-dependent Fe^{3+} accumulation in root tips on Pi-replete medium. Seeds were germinated as above (A). Root tips were monitored 3 d after transfer for Fe^{3+} accumulation by Perls staining coupled to diaminobenzidine (DAB) intensification (upper panel), or by Perls staining only (lower panel). Shown are representative images (n≥15). Scale bars, 50µm.

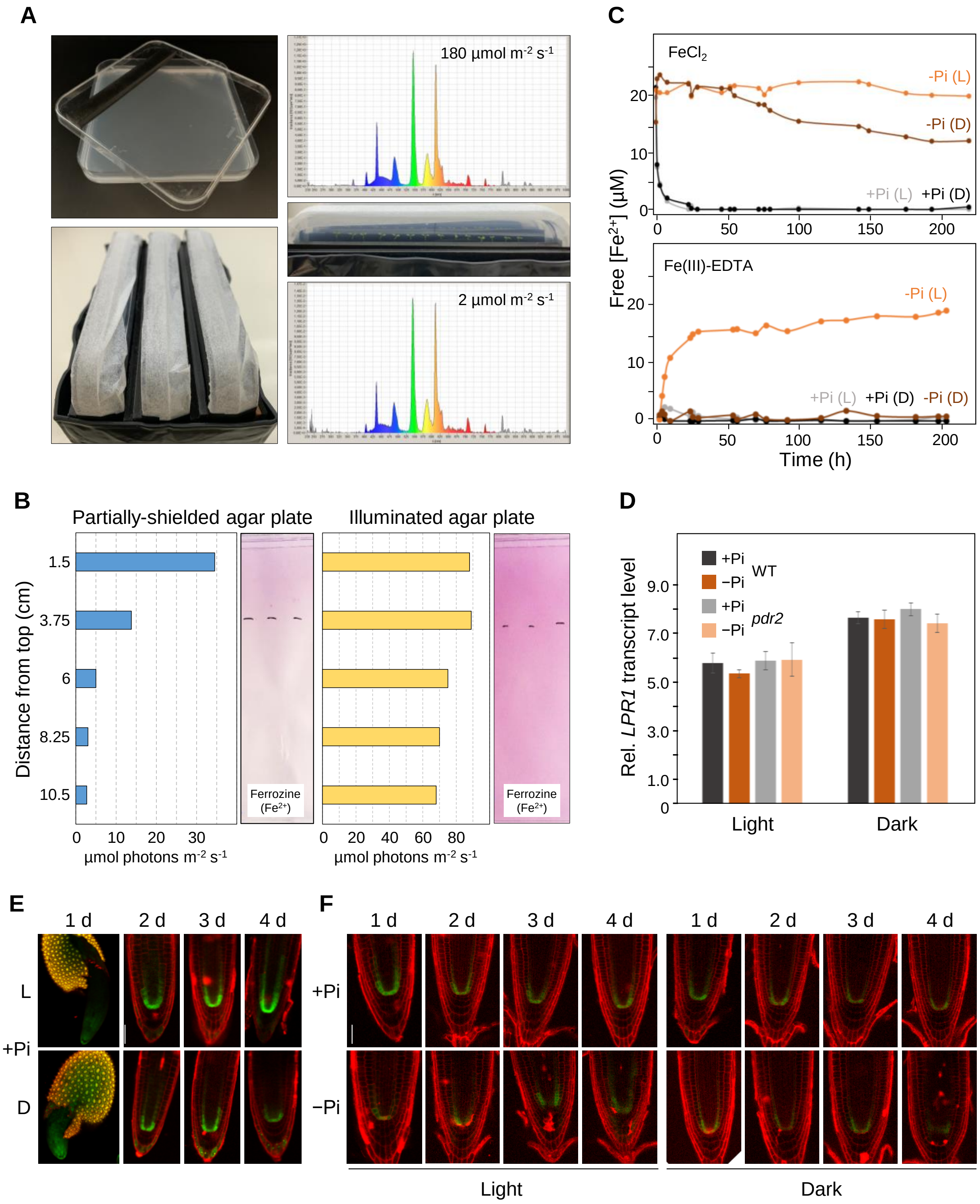

A
180 µmol m-2 s-1
2 µmol m-2 s-1
C
FeCl2
-Pi (L)
-Pi (D)
+Pi (L) +Pi (D)
Fe(III)-EDTA
-Pi (L)
+Pi (L) +Pi (D) -Pi (D)
Free [Fe2+] (µM)
Time (h)
B
Partially-shielded agar plate
Illuminated agar plate
Distance from top (cm)
1.5
3.75
6
8.25
10.5
Ferrozine (Fe2+)
µmol photons m-2 s-1
D
+Pi
−Pi
WT
+Pi
−Pi
pdr2
Rel. LPR1 transcript level
Light
Dark
E
1 d
2 d
3 d
4 d
L
+Pi
D
F
+Pi
−Pi
Light
Dark

Figure S5. Analysis of root growth on illuminated and partially light-shielded agar plates, Related to Figure 5

(A) Modified D-Root device[37] to allow for light-exposed shoot but light-shielded root growth in vertically oriented petri dishes. Black foamed plastic strip (117×12×6 mm) glued onto the lid (inside, 15 mm from top) of a 120 mm square petri dish (left upper panel). The shoot-root junction of transferred seedlings is placed between the agar and plastic strip surfaces. A black plastic box accommodates three assembled petri dishes separated by black foamed plastic sheets (left lower panel) such that the upper 15 mm of the dishes are exposed to ambient light (right center panel). Light spectra and intensities at illuminated shoots and light-shielded roots (right upper and lower panel, respectively). See also (B).
(B) Light intensities (bar graphs) and estimation of photo-Fenton Fe^{3+} reduction (Fe^{2+} ferrozine staining) in agar medium at different positions of vertically oriented, illuminated or partially light-shielded petri dishes. Light exposure causes conversion of Fe^{3+} (no complex formation with ferrozine, colorless) to Fe^{2+} (pink colored complex with ferrozine).
(C) Estimation of time-dependent Fe speciation (photo-Fenton Fe^{3+} reduction / Fe^{2+} autoxidation) by ferrozine assays of liquid +Pi of –Pi media supplemented with 25 µM $FeCl_2$ or 25 µM Fe^{3+}-EDTA, and exposed to illumination or darkness.
(D) Relative *LPR1* transcript levels (normalized to *UBC9*) in excised wild-type and *pdr2* root tips of seedlings germinated on +Pi agar (5 d) prior to transfer to illuminated (16 h L /8 h D) or partially light-shielded (dark) +Pi or –Pi medium (vertical agar plates). After 1 d, the gain in root tip growth was harvested for RNA preparations and qRT-PCR analysis (± SD; n = 3), no significant differences, Student’s t-test.
(E, F) Light-dependent expression of $LPR1_{pro}$*:GFP* in primary wild-type root tips. (E) Seeds were germinated for up to 4 d on illuminated (L) or completely dark-exposed (D) +Pi agar medium (vertical agar plates). (F) Seeds were grown as in (D) for up 4 d after transfer. Roots were counterstained with propidium iodide (red fluorescence) and GFP-derived fluorescence (green) was monitored. Shown are representative images (n≥15). Scale bars, 50 µm.

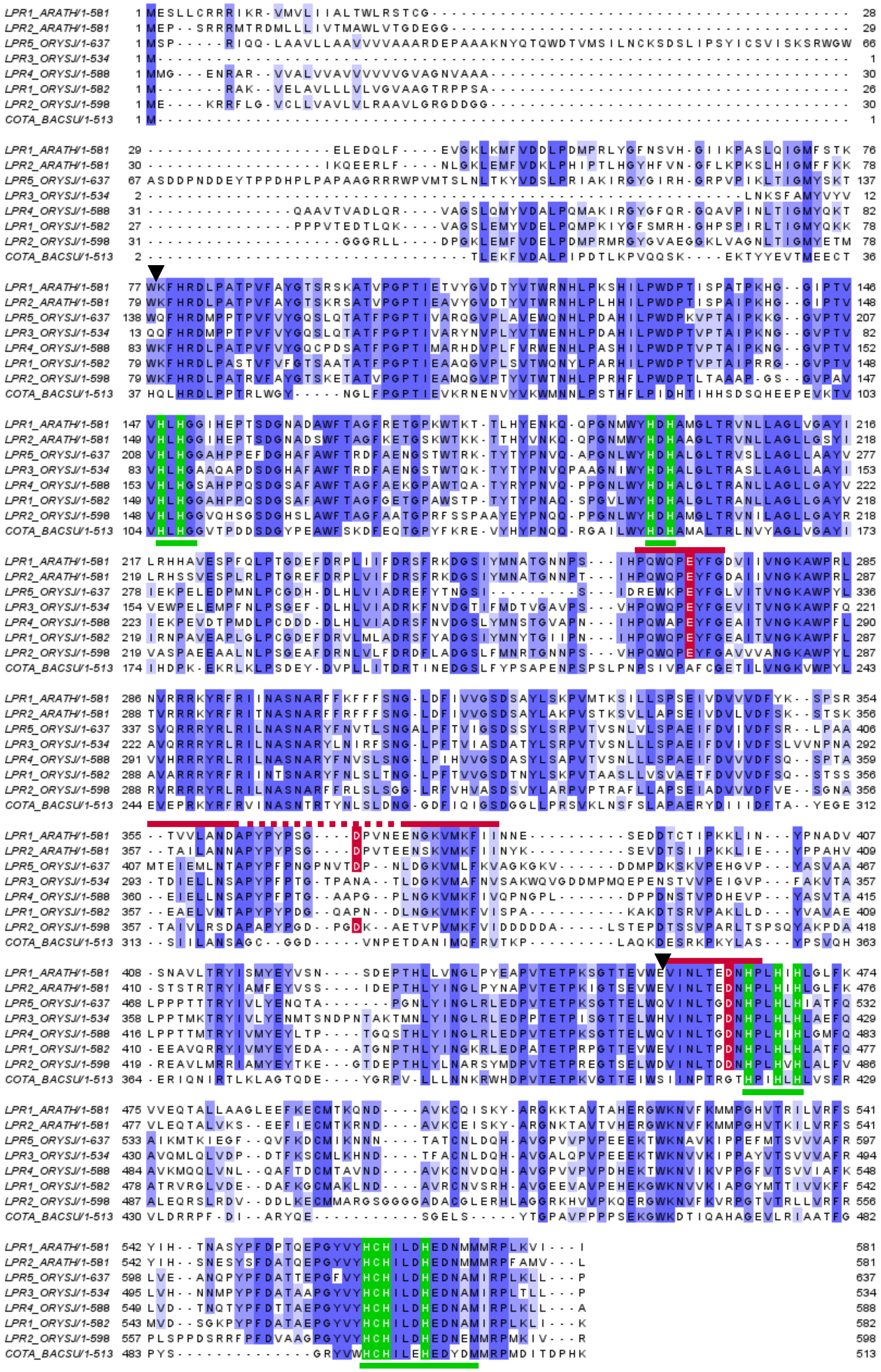

Figure S6. Alignment of primary structures of CotA and LPR1-like Arabidopsis and rice proteins, Related to Figure 6

Alignment of amino acid sequences of LPR1 and LPR2 (*Arabidopsis thaliana*), LPR1-5 (*Oryza sativa*) and CotA (*Bacillus subtilis*). Shades of blue reflect degrees of positional sequence identity. Invariant histidine/cysteine residues of the T1 and T2/T3 copper cluster are shaded in green. The four conserved copper binding motifs typical for the MCO family (HXHG, HXH, HXXHXH, and HCHXXXHXXXXM/L/F) are delineated by green lines below the alignment. Residues of the acidic triad (presumed Fe^{2+} binding site) are depicted in red (E269, D370 and D462 on LPR1). Red lines above the alignment indicate conserved motifs flanking each residue of the acidic triad. Black triangles depict the position of conserved phase-0 introns.

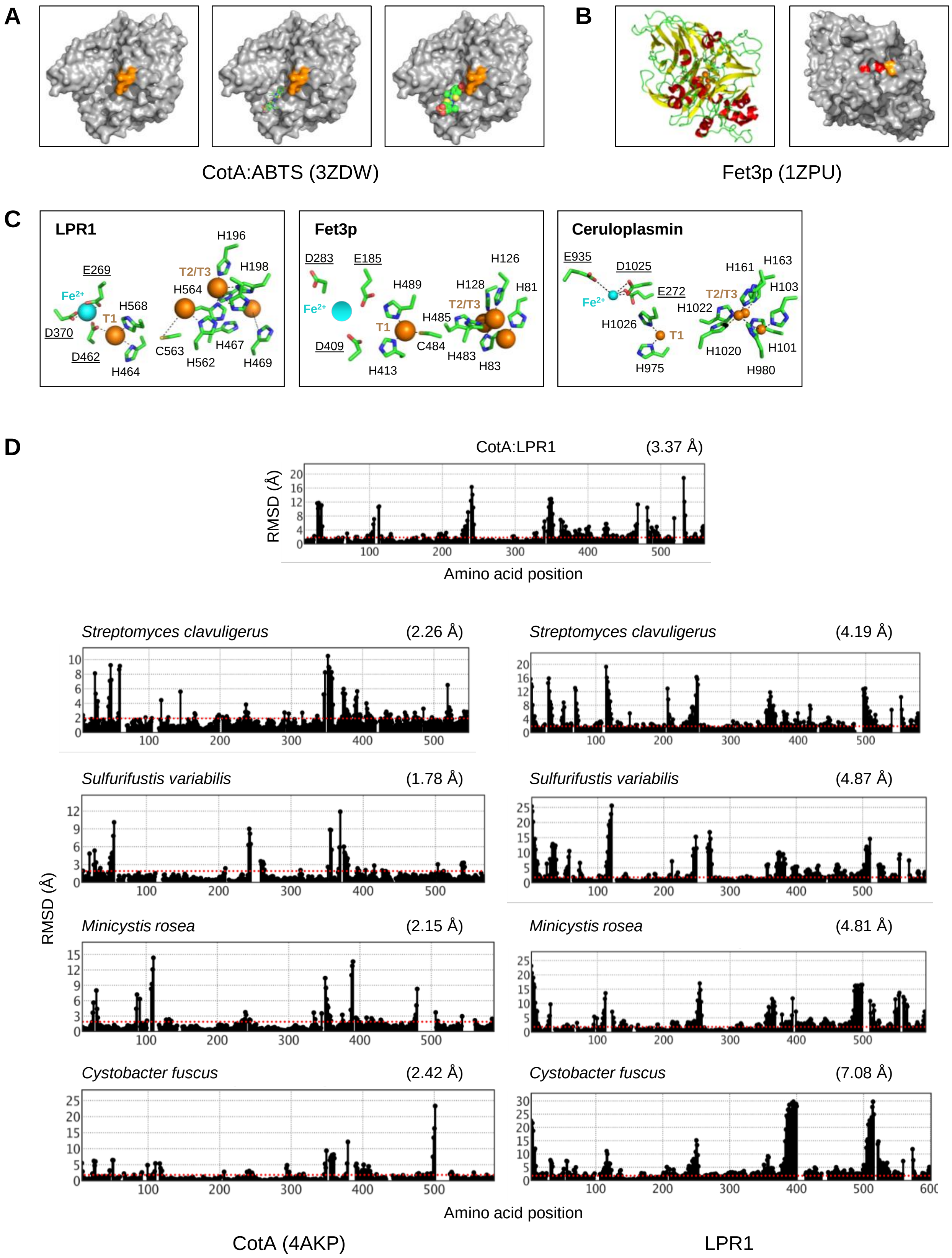

A
CotA:ABTS (3ZDW)
B
Fet3p (1ZPU)
C
LPR1
E269
Fe2+
H568
T1
D370
D462
H464
H196
H198
T2/T3
H564
C563
H467
H562
H469
Fet3p
D283
E185
H489
H126
H128
H81
T2/T3
Fe2+
H485
T1
C484
D409
H413
H483
H83
Ceruloplasmin
E935
D1025
H161
H163
E272
T2/T3
H103
Fe2+
H1022
H1026
T1
H975
H1020
H101
H980
D
CotA:LPR1
(3.37 Å)
RMSD (Å)
Amino acid position
Streptomyces clavuligerus
(2.26 Å)
(4.19 Å)
Sulfurifustis variabilis
(1.78 Å)
(4.87 Å)
Minicystis rosea
(2.15 Å)
(4.81 Å)
Cystobacter fuscus
(2.42 Å)
(7.08 Å)
Amino acid position
CotA (4AKP)
LPR1

Figure S7. Substrate-binding sites of CotA, LPR1, Fet3p, and ceruloplasmin, Related to Figure 6

(A) Surface representations of the CotA:ABTS complex (PDB: 3ZDW). The loop next to the substrate-binding site is highlighted (orange). Left panel: no ABTS. Center panel: ABTS (sticks). Right panel: ABTS (space filling).
(B) Experimental structure of Fet3p (PDB: 1ZPU). Left panel: Ribbon presentation (Cu ions as orange spheres). Right panel: Surface representation (acidic triad in red, loop in orange).
(C) Structural models of the Fe^{2+} (blue sphere) binding site, and the T1 and T2/3 Cu (orange spheres) sites in LPR1, Fet3p, and ceruloplasmin (PDB: 1KCW).
(D) Superposition of the experimental structure of *Bacillus* CotA (PDB: 4AKP) and the homology model of *Arabidopsis* LPR1 (top diagram). Superposition of LPR1-like MCO models of predicted proteins from *Streptomyces clavuligerus*, *Sulfurifustis variabilis*, *Minicystis rosea*, and *Cystobacter fuscus* with the experimental structure of CotA (diagrams on the left) and the homology model of *Arabidopsis* LPR1 (diagrams on the right). Plotted are the RMSD values (Å) for each corresponding amino acid residue. The average RMSD values of all residues are given above each diagram (parenthesis). The rather high average values are caused by the loop structures, which differ in length and shape (high RMSD peaks) whereas the superposition of defined secondary structure elements reveal low RMSD values (dotted red lines indicate the average RMSD values without loop structures).
