# Supplementary material for: Bacterial-type ferroxidase tunes iron-dependent phosphate sensing during Arabidopsis root development
Source: Curr Biol. 2022 May 23;32(10):2189–2205.e6. doi: 10.1016/j.cub.2022.04.005 (PMC9168544; doi:10.1016/j.cub.2022.04.005)
Supplement: Data S3. Alignment of LPR1-related acidic triad motif polypeptide sequences, related to Figures 6 and 7 — Exhaustive searches were conducted (NCBI; published genomes;46,47,57–60 One Thousand Plant Transcriptomes Initiative48) to identify polypeptide sequences related to Arabidopsis LPR1 (see STAR Methods; Data S2A, S2C, and S4). Aligned are the conserved motifs embedding the acidic triad of LPR1 relative to E269, D370, and D462 (highlighted in red). Hydrophobic residues are highlighted in grey. Proline residues of the first motif (E269), except at the start, and in the center of the variable linker (flanking D370) are highlighted in petrol blue. Glycine residues in the variable linker segment, and highly frequent or invariant residues in all three motifs are typed in bold face. Aligned are the LPR1-like motif sequences of select embryophytes (from NCBI), of streptophyte algae (identified in published genomes [highlighted in yellow] or by the 1KP project), and of bacterial phyla (from NCBI). Note, sequences for some streptophyte algae are truncated (xxx). Three sequences are too long for reasonable alignment (∗, extra peptide sequences on the right). [file mmc4.pdf]

	269	370	462
**Embryophytes**			
Arabidopsis thaliana (LPR1)	PQWQPEYFG	SRTVVLANDAPYPYPSGDPVNEEN----------GKVMKFII	INLTEDNHP
Gossypium hirsutum	PQWQPEYFG	TDEAVLANDAPYPYPSGDPVNELN----------GRVMKFTI	INLTEDNHP
Sorghum bicolor	PQWQPEYFG	GHEAELVNTAPYPYPDGDAPNHLN----------GKVMKFVV	INLTQDNHP
Medicago truncatula	PQWQPEYFG	TNTVILANDAAYPYPSGDPVNEAN----------SKVMKFYI	INLTDDNHP
Solanum lycopersicon	PQWQPEYFG	SKSVILANDAVYPYPSGDPVNEEN----------SKVMKFII	INLTEDNHP
Zea mays	PQWQPEYFG	APEAELVNTAPYPYPDGDAPNHLN----------GKVMKFVV	INLTQDNHP
Phalaenopsis equestris	PQWQPEYFG	TDSVVLLNDAAYPFPSGEEPDNFS----------GKVMKFFI	INLTEDNHP
Amborella trichopoda	PQWQPEYFG	SDSVILANDAPYPFPTGDRTDALS----------GTVMKFSI	INLTGDNHP
Picea sitchensis	PQWQPEYFG	TGEAILTNDAVYPYPSGDPVDHLNE---------SKVMKFVI	IDLTDDNHP
Selaginella moellendorfii	PQWQPEYFG	RDEVLLRNTAAFPFPGGDAVDSNN----------GVVMKFLV	INLTEDNHP
Physcomitrium patens	AQWCPEYFG	GSEVFLNNSGQAPYPEGDAAFSPES--------TRSVMKFIV	INLTPDAHP
Marchantia polymorpha	PNWCPEYFG	GTAVYLNNSAPAPFPSGDPDFSPPS--------TNSVMAFRV	INFTPDAHP
Anthoceros agrestis	PSWCPEYFG	GSVLYLNNSGPAPFPSGDPSFSPAG--------TTFILRFTV	INLTEDGHP
**Zygnematophyceae**			
Mesotaenium endlicherianum	PHWLPEYFG	GSEIVVQNTANFPFPDGDSVDGN----------TNVVMKFLV	INLSEDAHP
Spirogloea muscicola	PNWVPEFFG	HRQLLLKNDANTPYPGGDPVTVN----------SATIMKFVI	INLTDDMHP
Penium margaritaceum	PAWTPEYFG	GASIRVVNDGGSPYPDGDPPTPA----------QRQVMLFVV	INLTKDTHS
Penium exiguum 670	TTWIPENFG	GDKVYLTNDAPTPYPDGDPVPPS----------QQQIMLFKV	VNLTPDTHP
Penium exiguum 718	PFWFPEYFG	GAEVRVLNSAPAPYIDGDAPDRS----------QKHVMLFKV	VNLTPDYHP
Penium exiguum 850	PHWAPEYFG	GAQVRMLNSAPAPYIDGDDPDRS----------QKQVMLFRV	VNLTPDYHP
Staurodesums convergens 100	PVWVPEYLG	CNDVILANDAPAPYPGGDPVDNS----------TGLVMKFKL	INATPDAHP
Staurodesmus convergens 492	PVWVPEYLG	CNDVILTNDAPAPYPGGDPANQD----------TGTVMKFTI	INATPDAHP
Staurodesmus convergens 829	PNWEPEYFG	QVPNGGEVVLAAPYPGGDPPTTSN---------QQNVMLFKV	VNLTPDTHP
Staurodesmus omearii 466	PFWNPEYFG	GSSVVMQNSAPAPFPGGDVPATDN---------LINVMKFSV	INLTDDWHP
Staurodesmus omearii 234	PHWNPEYIG	GSVVRLTNDAAAPFSGGDAVTPE----------VANVMLFVV	INLTPDWHP
Cosmarium granatum 450	ESWQPESFG	DMAVVMSNTAPIPYPGGDDDSNDP---------LWHVMQFRF	INLTPDLHP
Cosmarium granatum 198	xxxxxxxxx	GMSVIVSNTAPIPYPGGDQDNDDP---------LRHVMFFKI	INLTPDAHP
Cosmarium tinctum	PHWLPEYFG	CADIIVTNDGNAPYPGGDPVDGD----------SSMVMRIAV	INTTPDNHP
Cosmarium subtumidum	PIWIPEYFG	CDDVILVNDAVAPYPGGDAPDRN----------TALLMRIFL	INLTEDAHP
Closterium lunula	PNWVPEFFG	GTAVTVINDAPTPFPGGDPNAPP----------CRYVMRFIV	xxxxxxxxx
Pleurotaenium trabecula	PTWVPEYFG	GSEIFLKNSAPAPYPEGDFPVEQG---------LQFVMKFVV	INLTEDWHP
Phymatodocis nordstedtiana 1	PYWVPEHFG	GTPIILFNTAPYPFPDGDIPQGED----------TKVMLFRV	INLTPDSHP
Cosmocladium_cf. constrictum	PHWVPEYFG	GSFVHLMNDAPAPFKGGEEPEWN----------QEEVMAFAV	INLTGDWHP
Roya obtusa	PVWDPEYFG	GTQILLSNSANAPYPGGDPPSVD----------TSQVLKFIV	INLTDDVHP
Planotaenium ohtanii	PHWEPEYFG	GDEIVMHNAANAPYPGGDPPTLD----------VQMVMKFVV	VNLTEDTHP
Xanthidium antilopaeum	PNWVPEYFG	GTSVILKNSAVAPYPDGDTPDTPN---------LQNVMKFIV	xxxxxxxxx
Nucleotaenium eifelense	PEWNPEFFG	CSDVLLVSTADYPFPDGDEPTGDLP----------HVMRFVL	INLTPDTHP
Phymatodocis nordstedtiana 2	PLWYPEYFG	GAEVRLTNNAQAPFIDGDQPVGD----------MKDVMLFVV	VNLTPDWHP
Micrasterias fimbriata	PNWNPEYIG	CTDVIMTNDAPAPYPGGPSPSGDE----------GLVMKFQL	INTTPDAHP
Entransia fimbriata	NYWIPEYFG	GDEFVVTNDAPAPYPGGGDNATAIRGEIGP----GVLWKFVV	INLTEDFHP
**Coleochaetophyceae**			
Chaetosphaeridium globosum	PKWAPEFFG	TRLIILANKAATPFPDGDAVTGQ----------AASVMEFRV	INTTPDAHP
**Klebsormidiophyceae**			
Klebsormidium nitens-1	PVWEPEFFG	GTEIVLTNRAAAPFPDGDAPGDNGQFGGT-----AELMKFIV	INFSGDAHP
Klebsormidium nitens-2	PKWVPETFG	GAEIIMSNDAAAPFPDGAPVFGVNGT--------ASVLKFIV	FNPTADSHP
**Chlorophyceae**			
Microspora tumidula	PFWVPEYFG	GTEIFLRNSGPDDVYDGISIPIVANP-----QTTGQVMRFNV	YNLTPDAHP
Carteria obtusa	YQNSPYAYG	SDYLYVWNEYDPFRMSSEVMWCY----------SHLLAKITF	LGQDEIQHP

**Firmicutes**			
Bacillus albus	PSVVPEFFG	GQSIILTNDAPAPFPNGEPPDPN----------LTQIMEFRV	YNTTPDTHP
Bacillus toyonensis	PSVVPEFFG	GQSIILTNDAPAPFPNGEPPDSN----------LTQIMEFRV	YNTTPDTHP
Bacillus smithii	TSIVPEFFG	GKNIIMTNDAPAPFPTGDPPNEN----------TGNVMQFRV	INLTEDTHP
Bacillaceae bacterium BZC4	TSIVPEFIG	GKNIIMTNDAPAPFPTGDPPDEN----------TSTVMQFRV	INLTEDTHP
Geobacillus sp. 44B	TSIVPEFIG	GKNIIMTNDAPAPFPTGDPPDEN----------TSTVMQFRV	INLTEDTHP
Paenibacillus thiaminolyticus	PSVVPEFFG	GQSIILTNDAPAPFPDGDPPSPD----------LAQIMEFRV	YNTTPDTHP
Neobacillus mesonae	TSIVPEFFG	GKRIIMTNDAPAPYPNGEPVNPD---------TFGQVMEFRV	INLTNEVHP
Bacillus methanolicus	TSIVPEFIG	GKNIIMTNDAPAPFPTGDPVDEN----------TSAVMQFRV	INLTTDTHP
Bacillus PAMC26568 2	TSVVPEFFA	GKRIIVTNDAPAPFPDGDPANAV-----------GTVMEFRV	INLTDDSHP
Peribacillus butanolivorans 1	TSIVPEFFG	EKNIIVKNDAPAPFPDGDPANAV-----------GIVMEFRV	INLTTDTHP
Bacillus amyloliquefaciens	TSIIPEFVG	GKSIIMTNDAPAPFPTGDPVDEN----------TGTVMQFSV	INLTDDSHP
Bacillus pseudomycoides	PSVVPEFFG	GQSIILTNDAPTPFPDGESPSED----------LRQIMQFRV	YNTTPDTHP
Virgibacillus sp. Bac330	VSIVPEFVG	GKTVVVRNNAPIPYPNGEAPNPE---------TVGVIMQFKV	INTTGDTHP
Bacillus sp. THAF10	TSVVPEFFG	GYEVFMTNDAPAPFPEGDLANEVY-----------EVMKFKV	INLTPDTHP
Bacillus horikoshii	TSIVPEFFG	GKRITFINDAPAPFPNGDAENEISD-----------VLQFRI	INLTPDTHP
Clostridium carboxidivorans	PYWMPEFFG	KSKIILKNYANAPYPMGDPPDPN---------TVGQIMQFTI	VNLTQDAHP
Clostridium drakei	PYWMPEFFG	KNKIILQNYANAPYPMGDAPDPN---------TTGQIMQFTI	VNLTQDAHP
Clostridium botulinum	PYWQPEFFG	GTKIILNNDANAPYPTGDAPDKD---------TTGQIMQFTV	VNLTMDSHP
Clostridium sp. Prevot 594	PYWQPEFFG	GTRIILNNDANAPYPTGDAPDKD---------TTGQIMQFTV	VNLTMDAHP
Clostridium scatologenes	PYWMPEFFG	KTKIILQNYANAPYPMGDAPDPN---------TTGQIMQFTI	VNLTQDAHP
Clostridium sporogenes	PYWQPEFFG	GTRIILNNDANAPYPTGDAPDKD---------TTGQIMQFTV	VNLTMDAHP
Brevibacillus laterosporus	PSVVPEFFG	GENIILTNDAPAPFPDGTPPSPD----------ISQIMEFRV	YNVTPDTHP
Bacillus freudenreichii	TSIVPEFVG	GENITMTNDAPTPFPSGSPPN----------DNTGTVMQFRV	INLTKDSHP
Thermincola potens	PSIVPEFFG	GQNIILTNDAPTPFPNGNPVDPNTDP-------VGQLMQFRV	INTTPDTHP
Paenibacillus durus	PSVVPEFFG	GKRITLTNNAPAPFPDGKPGNLNP-------NTTGMIMQFRV	INTTADTHP
Peribacillus simplex	TSIIPEFVG	GKYIIMTNDAPAPFPTGN-GNQIN-------ENTGTVMQFRV	INLTTDTHP
Peribacillus butanolivorans 2	PSIVPEFFG	GERIILTNSANAPFPDGTPPTPD----------LAQIMEFRV	YNQTVDTHP
Clostridium pasteurianum	PYWVPEFFG	GDKIILGNDASAPFPTGTPADP---------ETVGQIMQFTV	VNLTPDAHP
Clostridium pasteurianum 2	PYWVPEFFG	GDKIILGNDANAPFPTGTPADP---------ETVGQIMQFTV	VNLTPDAHP
Bacillus PAMC26568 1	PYWVPEFFG	GTTVILMNNANAPFPGGTPADP---------QTVGQIMQFTV	VNLTDDTHP
Clostridium novyi	PYWQPEFFG	GTKIILTNSANAPFSSIKAPNN---------ETVGQVMQFTV	VNLTVDTHP
Sporolactobacillus terrae	TSITPEFIG	GKNIIMTNDAPAPFPGGDKPDP---------NTVGQVMQFRV	INVTGNSHP
Lysinibacillus macroides	ASIVPEFFG	GQRIILHNTAPAVFPGGDAPDP---------RTTGIVMEFRV	LNITGQTHP
Virgibacillus dokonensis	VSIVPSFAG	GSTVVMRNNAPTPFPNGELPNP---------ETVGVIMQFKV	INTTGDTHP
Bacillus coagulans	PSVVPFFLG	GQTILLKNDLGPNADPAD--------------QTGDVMQFRV	ANTTAFTHP
Virgibacillus necropolis	PSISPFFIG	GKTITLKNDLGPNASPED-------------ETD-EVMQFDV	TNITGFTHP
Virgibacillus phasianinus	PSITPFFLG	GMKVILKNNLGPNADPED-------------ETD-EVMQFDV	TNITGFTHP
Bacillus filamentosus	PSITPAFLG	GKTITLKNDLGPNANPDD--------------NTGEVMQFKV	INITGFTHP
Bacillus glycinifementans	PSIVPGFCG	GQTITLKNRIGCGGEEADP------------ETDADIMQFRV	INAGRAIHP
Bacillus sp. NSP9	PSIVPAFCG	GKTITLKNRIGCGGQDADP------------ETDADIMQFRV	VNAGRAIHP
Bacillus sp. KH172YL63	PSFVPHFFG	GKSITLENHARAPFPNGPAPDP---------DTTGSVMQFRV	MNVTEESHP
Clostridium argentinense	PYWRPAFFG	DEKVLLLNVSEDSPLDK--------------ETTGQVMQFKI	VNITGGAHP
Bacillus globigii	PSIVPAFCG	GQSIVLANSEGCGGPANP-------------ESDANVMQFRV	INPTRGTHP
Bacillus atrophaeus	PSIVPAFCG	GQSIVLANSEGCGGPANP-------------ESDANVMQFRV	INPTRGTHP
Bacillus PAMC 26568 3	PSIVPAFCG	GQSIILANSEGCGGDVNP-------------ETDANIMQFRV	INPTRGTHP
Bacillus tequilensis	PSIVPAFCG	GQSIILANSAGCGGDVNP-------------ETDANIMQFRV	VNPTRGTHP
Bacillus subtilis (CotA)	PSIVPAFCG	GESIILANSAGCGGDVNP-------------ETDANIMQFRV	INPTRGTHP
**Chloroflexi**			
Thermogemmatispora sp. A3-2	PYWIPEFFG	GRTLLLTNSAPAPFPDGDPPDPA---------TTGRVMQIRV	ANLTEDAHP
Dehalogenimonas formicexedens	LYWSPEFFG	GTKLVLNNLGPDSPFGGDLNDQAVPS------TTGRVMQFVV	VNTTMDAHP
Ktedonobacteria bacterium	PYWIPEFFG	GRSFTLVNDAPAPYPGGSSPDPN---------TNGQIMQFRV	INTTPDAHP
**Bacterioidetes**			
Hymenobacter sp. DG25A	PSVLPEFFG	GQTIIITNNAAIPFPDGDPVDADDAP--------AQIMAFKV	YNLTEDAHP
Hymenobacter sp. DG25B	PSILPEFFG	GKTIILTNNAAIPFPNGDPVEADDA--------LAQIMAFKV	YNLTEDAHP
Flavisolibacter sp. 17J28-1	PSILPEFFG	GQTIIVHNDAATPFPNGDAVDPA---------TAGKVMAFRV	YNETEDAHP
Nibribacter sp. BT10	PSILPEFFG	GKTLILTNNARTPYPFGDDVDADDSP--------SQIMAFSV	YNLTPDAHP

Proteobacteria

Sulfurifustis variabilis	PSVLPEFFG	GRTVVLRNDAPTPFPDGDPVDPQ---------TTGVVMAFRV	INATGDAHP	
Cystobacter fuscus	PSVLPEFFG	GARLTLHNDAPVPFPAGDPLAPEDP--------TRDIMRFDV	YNVTADAHP	
Geobacter pickeringii	xxxxPEFFG	GTSITMRNNAPTPFPGGDPVAAA---------TTGRIMQFRI	INNTVDMHP	
Minicystis rosea	PFWTPEAFG	GTKIILRNDARAPFPAGDSPDPN---------TTGQIMQFRV	VNLTGDTHP	
Geobacter bemidjinensis	PYWNPEL-G	GK-WLLKNTAKAPYPSGESPDGN---------TEGRIMQFLI	VNLTADAHP	
Geobacter metallireducens 1	PSAVPEMFG	GKNVILYSDAPAPFPLGDPRNDYFP*KNGFGPNTRVLMRFKV	ANLTGDTHP	*QWNTAGNPANALT
Desulfocurvibacter africanus	PSISPEFFA	GQSFILHNNAFTPFQGLDVQTDPANDSQPLP----EIMLFRV	VNLTDDVHP	
Thiobacillus denitrificans	PIWNPEFFG	GH-YVLGNVGPDEPFPGLEDEFDAADPD----TTGQIMQFRV	YNATGDAHP	
Pleomorphomonas sp. SM30	LDLAPEFFG	GAGIVLVNRLAPAPYPGGGNAAVPE----------NVMAFRV	TNETVDTHP	
Desulfuromonas soudanensis 8	PIWNPEFFA	GTVVRILNTAPDAPFGGFPDVPADPG------TTGQVMQFVV	FNLTMDAHP	
Sorangium cellulosum So0157	PIWVPEFFG	GAEIVLLNLGPDAPFTGGPQTPADPA------TTGQVMQFRV	YNFTVDAHP	
Nitrosococcus halophilus	PIWNPEFFG	GEAIYLVNVGPDEPFGGGRPGVDFEPANPE--TTGQVMKFQV	YNFTEDAHP	
Nitrosospira multiformis	PIWNPEFFA	GR-HVLKNIGPDEPFGGGVPGTDFQMADPR--SSGLIMQFHV	YNMTEDAHP	
Sorangium cellulosum	PIWNPEFFG	GR-HILANVGPDAPFGGGVPGVDFDRADPD--STGQIMAFDV	LNLTADAHP	
Azoarcus sp CIB	PIWNPEFFG	GTELHLINEGPDEPFGGGTPGTDFDAADPD--TTGQVMKLVV	YNFTEDAHP	
Pseudomonas oryzae	PMWNPEFFG	VGNYILGNLGPDEPFGGGQPDLDFPPADPA--STGQVMEFRV	YNTTGDAHP	
Sandaracinus amylooyticus	PIWNPEVFG	GDTITLLNRGPDEAWGGPHAMPPQDPADPT--TTGRVMQLRV	ANTTDDAHP	
Desulfuromonas soudanensis 6	PFWIPEFVG	GQTLLLNNTARTPFPKGAPVDPK---------TTGQIMQIVV	VNLTADAHP	
Geobacter metallireducens 2	PFWIPEFFG	GKTVTLTNNAKAPFPSGMAADPQ---------TTAQIMQFRV	INTTGDAHP	
Anaeromyxobacter dehalogenans	PYWNPEFVG	GTTLVLKNVAKTPYPGGATPAGGL----------SQLMQLRI	ANLTADSHP	
Geobacter sp.M21	PYWNPEFLG	SGKWLLKNTAKAPYPGGAAPSGNIE---------GRIMQFVV	VNLTADAHP	
Syntrophobacter fumaroxidans	PYWVPEFVG	GQTLEMRNTARTPWVGGA-PVNGN--------TTGKVMQFRV	LNLTADAHP	
Dyella jiangningensis	APWVPEVFG	GNQLELMNDSLP------------------------LMRFDV	VNLTGDTHP	
Luteibacter pinisoli	APWVPEVFG	GGTVELMNDALP------------------------LMRFAV	VNLTEDTHP	
Frateuria aurantia	APWVPECHG	GQAVELRHQGQA------------------------IMQFRV	VNITGDAHP	
Bradyrhizobium sp. CCBAU	PSAVPEFFG	GDTVTLLNVGPAFDPFKGISADGFLFGD*ANNPVGDIMQFAV	FNFTADSHP	*IEAAN
Pseudomonas lalkuanensis	PTALAEFFG	GSRVIMKNLGGDTPFGGTFGDDLAPEDLFDDRQTDRVMAFDV	YNFTADAHP	
Pseudomonas stutzeri	PSIVPAFCG	GESIILANSAGCGGDVNPETDAN-------------IMQLRV	INPTRGTHP	

Deinococcus-Thermus

Deinococcus ficus	ERWEPEFFG	GQTLTVTNDAATPYSGKPDKAGGTPPLPE-------LLQFRV	INNTVDVHP
Deinococcus NW56	ERWVPEFFG	GKLLRLTNEAETPYTGLADRRGGTPPLPE-------LMRVRV	INNTADVHP

Actinobacteria

Streptomyces clavuligerus	PSVLPEFFG	GALIDLTNDAPVPFPDGDPVAPPA----------DRVLRFRV	YNTTDDAHP
Thermobispora bispora	PIWVPEFFG	GTELYLVNEGPDVPYRGDGSDRPADPA-----TTGQVMKFVV	HNHTVDAHP
Raineyella sp. CBA3103	GPWIPEYFG	GKTILLQNSNPPTPISNPAPNLS------------QVMQFRV	VNTTPDTHP
Georgenia sp. Z443	PIWNPEFFG	GN-HVLGNLGPDEPFGGGEPGDDFPAADPE--TTGRVMQIRV	YNTTGDAHP
Agromyces flavus	PIWNPEFFG	GD-HVLGNVGPDEPFGGGEPGVDFPLANPQ--TTGQVLAFRV	FNTTGDAHP
Streptomyces nodosus	PIWNPEYFG	GTELYLINEGPDVPYPGGVAGKDFTPADAR--TTGQVMKFTV	HNLTNDAHP
Micromonospora carbonacea	PIWNPEFFG	GTELYLVNEGPDEPFCGGAPGTDFPPADPR--TTGQVLKFVV	RNCTEDAHP
Streptomycetaceae	PIWNPEFFG	GTGLYLINEGPDEAYSGGTPVTDFTPANIT--TTGQVMKFAV	YNFTSDAHP
Streptosporangium roseum	PIWNPEFFA	GTEIYLINEGPDEPFNGQPGTFADPA------TTGQVMKFKV	YNFTADAHP
Streptomyces autolyticus	PIWVPEFFA	GTELYLINEGPDGPFQGGTPGTDFPAADVN--TTGQVMKFVV	DNRTMDAHP
Streptomyces gilvosporeus	PIWNPECFG	GTSLYLINEGPDKAFGGGKPGTEFAPADPA--TTGQVMKFVV	HNFTADAHP
Kitasatospora aqueofaciens	PIWNPEFFG	GTGLYLINEGPDEAYSGGTPVTDFTPANI--TTTGQVMKFAV	YNFTSDAHP
Streptomyces solisilvae	PIWVPEFFA	GTELYLINEGPDGPFQGGTPGTDFPPADVN--TTGQVMKFVV	DNRTMDAHP
Salinispora arenicola	PIWVPEFFA	GTDLYLINEGPDGPFLGGVPGVDFAPADVN--TTGQVMKLAV	DNRTDHAHP
Kutzneria albida	PIWNPESFG	GTELYLINEGPDMAFNGAADATPADPA-----TTGQVMKFVV	ANFTADAHP
Intrasporangium calvum	PVWNPEFFG	GE-WVLHNVGPDEPFRGGDFE-PADAA-----TTGQVLQFRV	YNTTGDAHP
Tessaracoccus	PIWNPEFFG	GD-YPLNNLGPDAPFGGSRFKAADPS------AAGRIMQFRV	YNATMDAHP
Arthrobacter citreus	TSILPEFVG	GKSIILKNDAPAPFPDGNAVDENT----------GTIMQFRV	INLTPDAHP
Pseudarthrobacter sp. 1	RPWAPEFFG	GSRVVLRNSARVPYPDGPESVAGGAIPLP------QVMQFTV	INTTEDAHP
Pseudarthrobacter sp. 2	RPWAPEFFG	GTKVVLTNDARTPFPDGPVAVRRGGVPLRE------IMQFSV	INTTVDAHP
Arthrobacter sp U41	RPWAPEFFG	GAKVVLTNDARVPFPGGARAVRRGGSPLPQ------IMQFSV	VNTTVDAHP
Streptomyces venezuelae	PIWNPEFFA	GTELYLINEGADEPFGRGEPGVAFPVADPA--TTGQVMKFVV	HNFTADAHP
Streptomyces broussonetiae	PIWNPEFFA	GTELFLVNEGADQPFGRGEPGVDFPVADPA--TTGQVMKFVV	HNFTVDAHP
Rhodococcus sp. WMMA185	PTVLDEFFG	GQQIILKNLGPELPFRGYVDPADPE----------NSMKLVY	YNTTGSAHP

Cyanobacteria

Calothrix brevissima	PSVIPEFGSG	GETLILNNFAGDGDSEPVPNDP---------ETTSQIMAFKV	YNTTRDSHP	
Calothrix sp. NIES-2100	PSVIPEFGAG	GETLILNNFAGGITSEPIPNNP---------DTTGQIMAFRV	YNTTRDSHP	
Gloeothece citriformis	PSVLPEF-FG	GRNVTLQNNAPGTFKRPIVVDP---------KTTGQIMQFQV	YNTTVDNHP	
Leptolyngbya sp.NIES-3755	NSISPEF-FG	GDRLTLRNFGSDEPFAGLDDQGNLK*APADPDTTGQIMQFVV	YNTTADAHA	*DDR
Nostoc commune HK-02	APWTPEF-LG	GEKIVWQNDATSPFPFGRANAVFNLSFPLP-----EIMQFTV	INLQPVAHP	
Nostoc sp. Loabaria	PSVVPNF-YG	GKEFIIRNFDDKADS----------------ELTGQIIKIAV	YNTTQEPHP	
Nodularia spumigena	QKLTPAV-VH	GKEFILRNYGPGADP----------------NTTGQIIKFTV	YNTTQYTHT	
Nostocales cyanobacterium	PPGVPNR-TG	GKEFILRNYGPGADP----------------GTTGQIMKFTV	YNPTQYAHS	

Archaea

Halopiger xanaduensis	PSIVPEFYG	GETVLLHNDAPSLYRGSLEDSDETKPLP-------EIMLVDV	ANNTAMSHP
Natronococcus occultus SP4	PSIVPEFYG	GETLLLHNAAPSLYRGTREESEETKPLP-------ELVLVDV	ANHTSMSHP
Halostagnicola larsenii	PSIVPQFYG	GETLLLHNDAPALYRGEADNPDDDLVPLP------EIMLFDV	VNRTGMSHP
Natrinema sp. YPL30	PSIVPQFYG	GQTLLLHNDAPSLYRGTLEKSEETQPLP-------EIMLVDV	ANYTGMSHP
Haloterrigena turkmenica	PSIVPQFYG	GETLLLHNDAPAQYRGGMSSSDDDIVSLP------EIMLVDV	ANRSAMSHP
Halorubrum sp. CBA1229	PSVVPQFYG	GETLLLHNDAPATYRGTSGIEADGAEPLP------EIMLVDV	ANFTGMSHP
Natrinema versiforme	PSIVPQFYG	GETLLLHNNAPAQYRGRTGLEDDDIVSLP------EIMLVDV	ANRSGMSHP
Halolamina sp. CBA1230	PSIVPQFFG	GQTLLVHNDARAPYRGPDINTGNQQPLP-------DVMLLDV	ANLTGMSHP
Halophilic archaeon DL31	PSIVPQFFG	GQTLLVHNDAVAPYRGPEINAGNQQPLS-------EVMRIDI	VNLTGMSHP
Halosimplex pelagicum	PSIVPQFFG	GQTLLLHNNAVAPYRGPDINSGDQQPLP-------EVMLVDV	ANLTGVSHP

Data S3. Alignment of LPR1-related acidic triad motif polypeptide sequences, Related to Figure 6 and Figure 7

Exhaustive searches were conducted (NCBI; published genomes[54-58,113]; One Thousand Plant Transcriptomes Initiative[59]) to identify polypeptide sequences related to Arabidopsis LPR1 (see STAR Methods, and Data S2A, S2C and S4). Aligned are the conserved motifs embedding the acidic triad of LPR1 relative to E269, D370, and D462 (highlighted in red). Hydrophobic residues are highlighted in grey. Proline residues of the first motif (E269), except at the start, and in the center of the variable linker (flanking D370) are highlighted in petrol blue. Glycine residues in the variable linker segment and highly frequent or invariant residues in all three motifs are typed in bold face. Aligned are the LPR1-like motif sequences of select embryophytes (from NCBI), of streptophyte algae (identified in published genomes [highlighted in yellow] or by the One Thousand Plant Transcriptomes Initiative[59]), and of bacterial phyla (from NCBI). Note, sequences for some streptophyte algae are truncated (xxx). Three sequences are too long for reasonable alignment (*, extra peptide sequences on the right).
